# A pan-cancer atlas of metabolic regulatory circuitries integrating multi-omic, immune, and clinical dimensions

**DOI:** 10.3389/fmolb.2026.1845099

**Published:** 2026-05-26

**Authors:** Higor Almeida Cordeiro Nogueira, Emanuell Rodrigues de Souza, Victor dos Santos Lopes, Enrique Medina-Acosta

**Affiliations:** Laboratório de Biotecnologia, Centro de Biociências e Biotecnologia, Universidade Estadual Do Norte Fluminense, Campos Dos Goytacazes, Rio de Janeiro, Brazil

**Keywords:** cancer metabolism, metabolic regulatory circuitries, metabolic reprogramming, omic-specific metabolic signatures, OncoMetabolismGPS, regulated cell death

## Abstract

**Introduction:**

Metabolic reprogramming is a central determinant of tumor progression, immune evasion, and susceptibility to regulated cell death. However, the integration of metabolic regulation across genomic, multi-omic, immune, and clinical dimensions remains insufficiently resolved, limiting mechanistic interpretation and translational application in cancer.

**Methods:**

We constructed a Pan-Cancer atlas of metabolic regulatory circuitries using OncoMetabolismGPS, a multi-omic analytical framework integrating transcriptomic, epigenomic, genomic, proteomic, phenotypic, immunological, and clinical data across 33 tumor types. Significant regulator–signature associations were identified and aggregated into omic-specific metabolic signatures, which were further assembled into regulatory circuitries defined by shared upstream regulators and downstream metabolic programs. Circuitries were classified as convergent or divergent based on the directionality of their associations across phenotypic, prognostic, and immune dimensions.

**Results:**

A total of 463,433 significant associations were identified after pathway and regulatory multi-mapping of 171,782 primary associations, yielding 241,415 omic-specific metabolic signatures and 24,796 metabolic regulatory circuitries. Divergent regulatory relationships predominated across tumor types, while a substantial fraction of interactions exhibited convergent patterns, particularly in immune-cold microenvironments. Transcript isoform–level variation and tumor stemness emerged as dominant axes of metabolic regulation, accounting for the majority of significant associations. Over 75% of signatures exhibited at least one shared regulatory interaction, supporting the existence of coordinated regulatory modules linking metabolic pathways to tumor phenotypes, immune states, and clinical outcomes.

**Discussion/Conclusion:**

These findings support a multi-layered regulatory architecture in which metabolic programs are modulated by non-coding RNA regulators in a context-dependent manner across cancers. The resulting atlas provides a mechanistically structured bioinformatic resource for decoding metabolic pathway regulation, supporting the systematic identification of regulatory circuitries linked to tumor phenotypes, immune microenvironments, and clinical outcomes, and offering a foundation for the prioritization of candidate diagnostic markers and context-dependent metabolic vulnerabilities in cancer.

## 1 Introduction

The persistence of cancer as a leading public health challenge reflects its profound biological complexity, and the coordinated interplay of hallmarks, including uncontrolled proliferation, immune evasion, resistance to cell death, and metabolic reprogramming (Hanahan, 2022; Hanahan, 2026). Among these hallmarks, metabolic reprogramming is distinctive in that it not only sustains the energetic and biosynthetic demands of proliferating cells (DeBerardinis and Chandel, 2016; Faubert et al., 2020) but also modulates other hallmarks—particularly immune evasion and resistance to regulated cell death (RCD) mechanisms triggered by metabolic stress (Dias et al., 2024; Galassi et al., 2024; Mao et al., 2024).

Metabolic reprogramming plays a central role in immunosuppression, either by limiting essential nutrients to immune cells or through the accumulation of immunosuppressive metabolites such as lactate and kynurenine (Biswas, 2015; Galassi et al., 2024). Metabolic plasticity enables cancer cells to resist specific RCD pathways, such as apoptosis and ferroptosis, and more recently described metabolic forms of cell death, such as cuproptosis and disulfidptosis, through adaptations including the Warburg effect, glutamine dependence, or lipid remodeling (Mao et al., 2024; Dalseno et al., 2025). These adaptations generate metabolic dependencies that constitute therapeutic vulnerabilities exploitable through pathway-specific inhibition (Mao et al., 2026).

The metabolic alterations underlying metabolic reprogramming arise from (epi)genomic and transcriptomic modifications in genes encoding enzymes and their non-coding RNA regulators across multiple metabolic pathways (Sinkala et al., 2019; Mahe et al., 2023; Safi et al., 2023). However, despite the availability of extensive multi-omic datasets, systematic integrative analyses linking metabolic genes, non-coding regulators, tumor phenotypes, prognosis, and tumor immune context remain limited (Chakraborty et al., 2024; Hayes et al., 2024).

Existing multi-omic studies have characterized isolated aspects of tumor metabolism, immune remodeling, or RCD (Wang et al., 2023; Li Q. et al., 2024; Wang et al., 2025), yet no current framework resolves how these processes are jointly organized into coherent regulatory architectures across molecular, phenotypic, immune, and clinical layers. Such an integrative framework is strictly necessary because isolated, single-layer analyses cannot capture the multi-dimensional dependencies that dictate tumor survival and immune evasion. A structured pan-cancer approach is required to translate massive, disparate metabolic datasets into actionable, pathway-level targets. To address this gap, we introduce the concept of metabolic regulatory circuitries—paired metabolic and regulatory modules whose convergent or divergent associations encode the directionality of metabolic control within specific tumor contexts.

Building on our previous work on the multi-omic construction of RCD signatures (Rodrigues de Souza et al., 2025), we now extend this strategy to the metabolic domain by integrating enzyme-coding genes and their non-coding regulators across 64 pathways and seven metabolic categories from the Kyoto Encyclopedia of Genes and Genomes (KEGG) (Kanehisa and Goto, 2000). This analysis encompasses 33 cancer types from The Cancer Genome Atlas (TCGA) (Cancer Genome Atlas Research et al., 2013). This foundation enables the systematic derivation of multi-omic, phenotypic, and clinical relationships that underpin metabolic reprogramming at scale.

In this study, we introduce the concept of omic-specific metabolic signatures, defined as multidimensional units that integrate metabolic-pathway context with molecular, phenotypic, prognostic, immune, and clinical attributes within each tumor type. These signatures capture coordinated cross-layer behavior rather than a single dimension (e.g., simple co-expression), embedding multi-omic and clinical information into structured metabolic states. This formulation enables the systematic interpretation of multi-omic associations and addresses a central unresolved question in cancer metabolism: how metabolic programs, their non-coding regulators, and their phenotypic and clinical consequences are jointly structured across tumor types.

Using this integrative foundation, we developed OncoMetabolismGPS, a multi-omic analytical framework that operationalizes these metabolic signatures, maps their shared upstream regulatory interactions, and classifies each regulator–signature pair as convergent or divergent across molecular, phenotypic, immune, and clinical dimensions. This strategy reconstructs a Pan-Cancer atlas of metabolic regulatory circuitries, enabling the systematic identification of aligned *versus* opposing regulatory influences within metabolic pathways. The importance of this atlas lies in its capacity to transition cancer metabolism research from isolated single-gene observations to an integrated, systems-level understanding of regulatory architecture. By comprehensively mapping these circuitries across 33 tumor types, the atlas provides a critical translational resource to uncover how non-coding RNAs coordinate metabolic dependencies, immune evasion, and patient prognosis, thereby accelerating the discovery of context-specific metabolic vulnerabilities for targeted therapeutic interventions.

**FIGURE 1:**
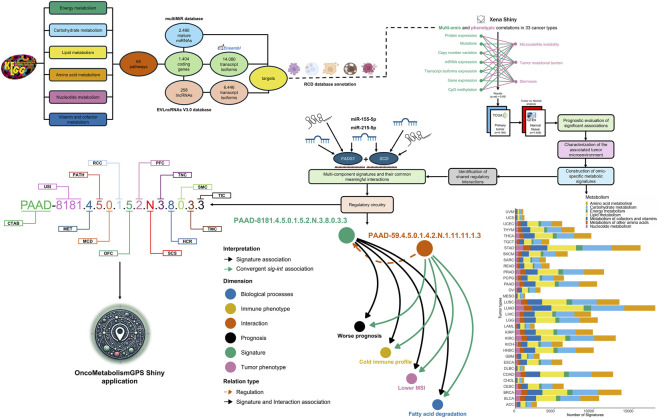
Overview of the OncoMetabolismGPS framework for the identification, integration, and interpretation of multi-omic metabolic signatures in cancer. The workflow begins by selecting enzyme-coding genes from KEGG-defined metabolic pathways, together with their experimentally validated miRNA and lncRNA regulatory interactions (sourced from miRTarBase and EVlncRNAs 3.0). These components are consolidated into a core set of molecular targets, forming the ‘analytical unit targets’. These targets are further annotated for their involvement in regulated cell death (RCD) mechanisms prior to downstream association testing. Subsequently, multi-omic, phenotypic, and clinical data across 33 TCGA tumor types are integrated. Significant molecular associations are then evaluated for clinical impact and contextualized with immune microenvironment features inferred *via* cell deconvolution. Based on these integrations, omic-specific metabolic signatures are constructed for each cancer type—defined by shared metabolic function, phenotypic directionality, prognostic coherence, and immune compatibility. For each signature, shared upstream regulators are identified to map integrated regulatory circuitries, which are then classified as convergent or divergent based on their cross-dimensional behavior. Every signature receives a standardized 14-token nomenclature encoding its tumor type, omic layer, metabolic pathway, RCD involvement, phenotypic class, prognostic direction, and immune profile. Collectively, these components comprise the OncoMetabolismGPS framework. Serving as a multi-omic “Guided Positioning System,” it systematically organizes metabolic signatures from the molecular to the clinical level, enabling the discovery of regulatory circuitries that link metabolism to clinical and immune outcomes. All resulting signatures, interactive networks, and evaluation metrics are accessible *via* the OncoMetabolismGPS Shiny application (Section 3.10).

Figure 1 provides an overview of the analytical pipeline, including data integration, association analyses, signature generation, regulatory evaluation, and circuitry classification.

Finally, we establish a standardized nomenclature system, and we implement all findings in the OncoMetabolismGPS Shiny application (https://oncometabolismgps.be/). The atlas provides an interactive environment for visualizing metabolic signatures, interrogating regulatory circuitries, and generating tailored signature sets across molecular, phenotypic, and immunological domains.

Together, these components establish a comprehensive framework for reconstructing metabolic regulatory circuitries across cancers, enabling systematic exploration of how metabolic pathways interface with non-coding RNA regulation, tumor phenotypes, immune profiles, and clinical outcomes.

From a bioinformatics perspective, this problem can be understood as one of representation—how to organize multi-omic associations into coherent, interpretable models of pathway-level regulation across tumor contexts.

In this context, the proposed framework enables systematic reconstruction of molecular pathway organization across multi-omic layers, linking regulatory architecture to phenotypic and clinical outputs and supporting the prioritization of pathway-level biomarkers and candidate diagnostic targets across tumor contexts. Beyond its analytical utility, this Pan-Cancer atlas provides the broader scientific community with a systems-level resource to interpret how metabolic regulatory architectures shape tumor behavior, immune evasion, and patient-relevant phenotypes. By making these multi-omic circuitries structurally accessible, this framework is intended to accelerate the identification of context-dependent metabolic vulnerabilities, providing a robust foundation for future basic and translational investigations with clear therapeutic relevance.

## 2 Methods

The following sections describe the data sources, integration strategy, and computational procedures used to construct and analyze the metabolic regulatory circuitries.

### 2.1 Identification of metabolism-related coding and non-coding RNAs

We retrieved 1,404 human enzyme-coding genes involved in anabolic and catabolic reactions across 64 KEGG-defined metabolic pathways, grouped into seven major metabolic categories: carbohydrate metabolism, lipid metabolism, energy metabolism, amino acid metabolism, metabolism of other amino acids, nucleotide metabolism, and metabolism of cofactors and vitamins (Supplementary Table S1). Gene selection was based on the Kyoto Encyclopedia of Genes and Genomes using the KEGGREST package (Kanehisa and Goto, 2000; Tenenbaum, 2017).

For these enzyme-coding genes, we identified 2,490 miRNAs (Supplementary Table S2) and 258 lncRNAs with experimentally validated regulatory interactions (Supplementary Table S3). miRNA-mRNA interactions were obtained from miRTarBase (Cui et al., 2025), and lncRNA-mRNA interactions from EVlncRNAs 3.0 (Zhou et al., 2024). For each gene and regulatory RNA, transcript isoform identifiers were retrieved from Ensembl (GRCh38. p13) (Durinck et al., 2005), totaling (20,526) annotated isoforms (Supplementary Tables S4, S5).

### 2.2 Annotation of regulated metabolic cell death mechanisms

To determine the involvement of molecular targets in RCD mechanisms with metabolic dependency, we performed functional annotation using a manually curated knowledge base of RCD (RCD) pathways (Wang et al., 2024). Targets were classified according to their participation in ferroptosis, apoptosis, necroptosis, parthanatos, and other metabolic cell death programs (Supplementary Table S6).

### 2.3 Multi-omic, phenotypic, and clinical integration across 33 cancer types

To further characterize the molecular targets, we integrated multi-omic, phenotypic, and clinical data from TCGA *via* UCSC Xena, covering 33 distinct cancer types (Li S. et al., 2024) (Supplementary Table S7). The omic layers analyzed included bulk-RNA gene expression, transcript-isoform expression, miRNA expression, CpG methylation, copy number variation (CNV), somatic mutation, and protein expression (RPPA array), as comprehensively detailed in the Supplementary Material (Method C). In parallel, we incorporated clinical and phenotypic data, including immunogenomic features with potential impact on immunotherapy response, such as microsatellite instability (MSI), tumor mutational burden (TMB), and stemness indices reflecting tumor plasticity and self-renewal capacity, which have been linked to immune evasion, cold tumor microenvironments, and reduced immunotherapeutic efficacy.

The analyses were conducted using TCGA cohorts comprising 9,185 tumor samples across 33 cancer types and GTEx cohorts including 7,414 samples from 30 primary sites (Supplementary Table S8). All statistical evaluations were performed within tumor-specific cohorts, such that each association test was restricted to the samples available for the corresponding cancer type and omic layer. As a result, sample sizes varied across analyses due to differences in data availability and dataset-specific filtering procedures. This approach ensured that all statistical inferences were derived from biologically homogeneous tumor cohorts. A detailed summary of sample counts per cancer type and omic layer is provided in Supplementary Table S9.

### 2.4 Computational pipeline for integrative association testing

We developed an R-based pipeline to systematically evaluate associations between molecular features and tumor phenotypes. Approximately 2.94 million pairwise association tests were performed across cancer types and omic layers.

Association analyses were stratified by cancer type and omic layer. Multiple-testing correction was applied using the Bonferroni method, and associations with adjusted p-values <0.05 were significant.

Pairwise associations between molecular features and tumor phenotypes were computed using Spearman rank correlation, selected for its robustness to non-normal distributions and heterogeneous scaling across omic layers. All association tests were performed independently within each cancer type and omic layer, ensuring that statistical relationships were evaluated in biologically homogeneous contexts and avoiding cross-tissue or cross-platform confounding.

For each association, both the correlation coefficient (*ρ*) and corresponding p-value were calculated.

Multiple testing correction was applied using the Bonferroni method across all pairwise associations within each cancer type and omic layer, providing a conservative control of false-positive rates. All statistical analyses were implemented in R.

### 2.5 Prognostic evaluation of significant associations

Molecular features significantly associated with tumor phenotypes were subsequently evaluated for prognostic value only in the same tumor type and omic layer where the association had been detected. For example, if the expression of a gene was significantly correlated with increased stemness in lung adenocarcinoma (LUAD), its prognostic evaluation was restricted to that specific tumor-omic context.

For each selected instance, we applied univariate Cox proportional hazards regression to estimate the hazard ratio of clinical outcomes, complemented by log-rank tests to compare survival distributions between stratified groups in clinical endpoints, including OS, DSS, DFI, and PFI.

### 2.6 Characterization of the tumor immune microenvironment

To characterize the tumor microenvironment linked to the omic layers of significant targets, we analyzed correlations between molecular features and infiltrating immune cell populations.

Relative abundance estimates for 29 immune cell types were derived using the CIBERSORT and xCELL deconvolution method applied to TCGA transcriptomic profiles (Supplementary Table S10) (Aran et al., 2017; Steen et al., 2020). Each cell type was categorized into anti-tumor, pro-tumor, or dual functional groups based on literature evidence.

Omic layers were then classified according to the predominant associated immune infiltration pattern. For refined categorization, we focused on five immune cell subsets - CD8^+^ T cells, NK cells, regulatory T cells (Tregs), M1 macrophages, and M2 macrophages - widely recognized as markers of hot *versus* cold tumors. Associations dominated by effector infiltrates (CD8^+^, NK, M1) were classified as hot, those dominated by suppressive infiltrates (Tregs, M2) as cold, and intermediate or mixed profiles as variable (Supplementary Material–Methods A and B).

### 2.7 Construction of omics-specific metabolic signatures

To integrate multi-omic associations into biologically interpretable units, we constructed omic-specific metabolic signatures for each molecular class (mRNAs, miRNAs, lncRNAs, and transcript isoforms). A metabolic signature was defined as a set of targets that jointly satisfied a set of four hierarchical criteria, ensuring both mechanistic coherence and translational relevance. Signature construction was performed independently within each cancer type to preserve tumor-context specificity.

First, candidate elements had to share functional grounding in metabolism, evidenced by (i) participation in the same KEGG metabolic pathway and (ii) annotation to the same metabolism-RCD mechanism (RCD database). Second, all elements in a signature were required to exhibit concordant associations with tumor phenotypes (MSI, TMB, and/or stemness indices) within a given tumor type and omic layer. Third, these associations needed to display consistent prognostic directionality across survival outcomes (OS, DSS, DFI, PFI), as determined by Cox proportional hazards models and validated by log-rank testing. Finally, signatures were required to exhibit immunological compatibility, meaning that all components were embedded in tumor microenvironments characterized by the same immune state (hot, cold, or variable), as inferred from immune infiltration profiles.

Elements that met all four criteria were grouped into multi-component signatures when they shared identical biological and clinical association profiles across dimensions. When a candidate element had no additional partners meeting these criteria, it was kept as a single-component signature, preserving isolated yet potentially critical metabolic features.

Because some metabolic genes and their regulatory RNAs participate in multiple pathways and cell death processes, the same genetic element could appear in more than one signature. This controlled redundancy is a deliberate feature of the framework: it captures the multifunctionality and pleiotropic roles of metabolic regulators rather than collapsing them into a single assignment. By allowing biological context to guide grouping rather than forcing mutual exclusivity, the resulting signatures more accurately reflect the true network architecture of tumor metabolism.

It is important to distinguish the levels at which multi-omic integration operates in this framework. The analytical pipeline is multi-omic, as associations are systematically evaluated across multiple molecular layers (gene expression, transcript isoforms, miRNAs, lncRNAs, methylation, CNV, mutation, and protein expression). However, the resulting metabolic signatures are defined as mono-omic entities, each constructed within a single omic layer to preserve mechanistic coherence and avoid conflating distinct regulatory modalities.

Integration across omic layers is achieved at the level of regulatory circuitries, where signatures derived from different molecular layers—such as enzyme-coding gene signatures and non-coding RNA signatures—are paired through experimentally validated regulatory interactions. This design enables cross-omic coupling while maintaining the interpretability and biological specificity of individual signatures.

### 2.8 Identification of shared regulatory interactions

To determine common regulatory control, experimentally validated miRNA-mRNA and lncRNA-mRNA interaction tables were intersected across all members of each signature. A regulator was kept only if it interacted with every component of the signature. The same logic was applied inversely for miRNA-based signatures to identify shared coding targets. The algorithm intersected the interactions of all components within each signature, keeping only regulators or targets common to all members. For example, in a signature composed of two enzyme-coding genes initially linked to miR-21, miR-155, and miR-35, only miR-21 interacted with both genes and was therefore recorded as the validated shared regulator. Similarly, for miRNA-based signatures, shared coding gene targets were identified.

In a limited number of cases, the same biological regulator–signature pair is instantiated twice with inverted primary–interaction roles, reflecting primary-signature indexing rather than biological duplication. These swapped circuitries are retained by design and explicitly annotated in the supporting datasets; a detailed methodological clarification and computational audit procedure are provided in Supplementary Note 1 (Supplementary Material).

### 2.9 Convergence *versus* divergence analysis of regulatory circuitries

For each cancer type, we evaluated whether the signature and its shared regulator showed aligned (convergent) or opposing (divergent) associations with tumor phenotypes (MSI, TMB, stemness), survival outcomes (Cox and log-rank analyses), and immune microenvironment classifications.

Interactions were convergent when both the signature and its shared regulator showed the same direction of association (e.g., both protective in OS or both enriched in anti-tumor microenvironments). Conversely, they were classified as divergent when associations were opposite, indicating inverse directional relationships between regulatory and metabolic components.

### 2.10 Signature nomenclature system

We designed a standardized signature nomenclature system to ensure traceability and clarity across computational analyses and visual outputs. Every signature was assigned a unique alphanumeric identifier incorporating: (i) tumor type, (ii) omic layer, (iii) metabolic pathway category, (iv) metabolic cell death involvement, (v) presence of shared regulatory interactions, (vi) phenotypic association class (MSI, TMB, TSM), (vii) prognostic classification across clinical outcomes, and (viii) tumor microenvironment and immune infiltration profiles. A full description of all encoding rules, scoring weights, and rank calculations is provided in the Supplementary Material (Method C).

## 3 Results

### 3.1 Molecular targets identified in metabolic processes and annotation in metabolic cell death mechanisms

We retrieved 1,404 unique enzyme-coding genes functionally associated with catalytic reactions distributed across 64 KEGG-defined metabolic pathways encompassing anabolic and catabolic processes. These pathways were grouped into seven major metabolic categories: carbohydrate metabolism, lipid metabolism, energy metabolism, amino-acid metabolism, metabolism of other amino acids, nucleotide metabolism, and metabolism of cofactors and vitamins (Supplementary Table S1). For these enzyme-coding genes, we identified 2,490 mature microRNAs (miRNAs) and 258 long non-coding RNAs (lncRNAs) with experimentally validated regulatory interactions (Supplementary Tables S2, S3). For each enzyme and regulator, 20,526 transcript isoform identifiers (GRCh38. p13) were retrieved from Ensembl (Supplementary Tables S4, S5).

Among these targets, 247 unique enzyme-coding genes were annotated to regulated metabolic cell death forms, including alkaliptosis, apoptosis, autophagy-dependent cell death, cuproptosis, entotic cell death, ferroptosis, lysosome-dependent cell death, necroptosis, oxeiptosis, parthanatos, and pyroptosis (Supplementary Table S6).

### 3.2 Multi-omic and phenotypic associations across 33 cancers

We developed an R-based computational pipeline to perform approximately 2.94 million association tests across multiple omic layers—bulk-RNA gene expression, transcript-isoform expression, miRNA expression, CpG methylation, copy number variation (CNV), somatic mutation, and protein expression—evaluating their relationships with three phenotypic variables: microsatellite instability (MSI), tumor mutational burden (TMB), and tumor stemness (TSM). The analyses included molecular targets spanning enzyme-coding genes, miRNAs, lncRNAs, and transcript isoforms, examined across 33 TCGA cancer types.

A total of 171,782 associations met the significance threshold (adjusted p < 0.05) across omic layers, phenotypic measures, and tumor types (Dataset S1). Transcript-isoform expression accounted for 51.7% of these associations, followed by gene expression (15.4%), CpG methylation (14.7%), somatic mutation (7.5%), miRNA expression (6.3%), and CNV (4.4%). Significantly fewer correlations were observed for protein expression. By phenotypic variable, TSM contributed 72.1% of associations, TMB 14.3%, and MSI 13.6%.

After annotation to metabolic pathways, metabolic cell death mechanisms, and regulatory interactions, the number of associations expanded—through legitimate multi-mapping—to 463,433 entries (Dataset S2), reflecting the intrinsic multifunctionality of metabolic genes and their non-coding RNA regulators.

### 3.3 Prognostic relevance and tumor microenvironment profiles of significant associations

Operationally, prognostic relevance was strictly defined as any association possessing at least one clinically significant outcome across the evaluated survival metrics (OS, DSS, PFI, or DFI). An association met this criterion if it exhibited a defined “Protective” or “Risky” hazard classification in univariate Cox regression models, and/or presented a statistically distinct worst-prognosis group *via* Kaplan-Meier survival analysis. By applying this comprehensive requirement (and strictly excluding any non-significant or absent clinical data), out of the 463,433 computed associations, 186,605 (40.3%) exhibited prognostic relevance. Among these prognostic associations, TME classes were distributed as dual 50.5%, anti-tumoral 23.8%, pro-tumoral 17.4%, and non-significant 8.3%. Immune-phenotype classification revealed a predominance of cold profiles (81.6%), followed by hot 12.3% and variable 6.0% (Dataset S2).

### 3.4 Construction of omics-specific metabolic signatures

To integrate multi-omic and phenotypic associations into biologically interpretable entities, we constructed omic-specific metabolic signatures within each cancer type. Each signature is mono-omic, comprising one or more elements from the same omic layer—enzyme-coding genes, miRNAs, lncRNAs, or transcript isoforms—that (i) share a common KEGG-defined metabolic pathway and the same metabolic cell death mechanism; (ii) exhibit concordant associations with tumor phenotypes and prognostic outcomes across all survival endpoints; and (iii) align with the same immune microenvironment class.

Elements displaying fully concordant profiles across molecular, phenotypic, prognostic, and immune dimensions were grouped into composite signatures, whereas unmatched gene attributes were kept as single-member signatures. Because a substantial proportion of enzyme-coding genes take part in multiple pathways or cell death mechanisms, individual gene attributes may recur in distinct context-specific signatures. We identified 241,415 signatures across 33 tumor types (Dataset S3).

**FIGURE 2:**
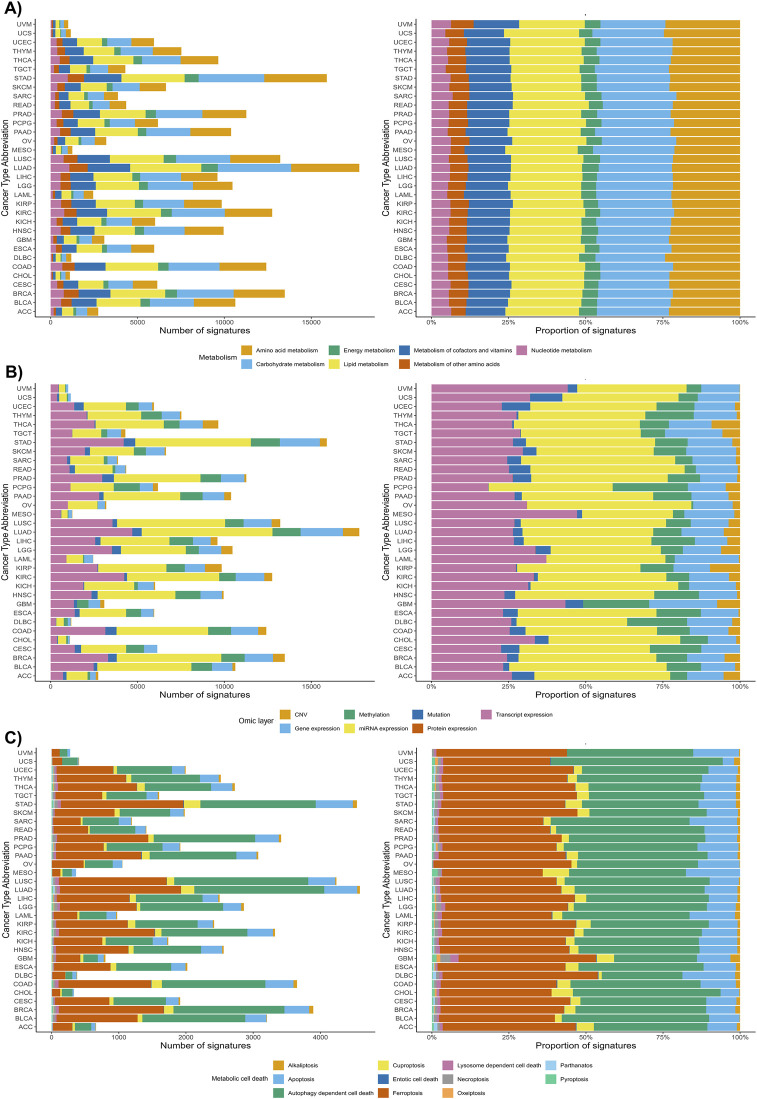
Distribution of omic-specific metabolic signatures across tumor types, molecular layers, and metabolic contexts (n = 241,415 signatures; Dataset S3). For each category (panels a, b, and c), data are presented in two columns: the left panels display absolute signature counts, and the right panels display relative proportions per cancer type. Notably, the proportional distribution of signatures per omic layer, metabolic category, and metabolic cell death mechanism is highly conserved across tumor types (statistically confirmed by near-perfect cross-cohort Pearson correlations and minimal Cramer’s V effect sizes), with minor exceptions driven primarily by extremely low signature counts in rare categories. **(A)** Distribution of signatures by metabolic category across 33 TCGA tumor types: carbohydrate metabolism (57,563), lipid metabolism (56,870), amino acid metabolism (53,422), metabolism of cofactors and vitamins (33,485), metabolism of other amino acids (14,186), nucleotide metabolism (13,831), and energy metabolism (12,058). **(B)** Distribution by omic layer: miRNA expression (102,272), transcript-isoform expression (65,590), gene expression (31,694), CpG methylation (25,400), CNV (8,608), mutation (7,827), and protein expression (24). **(C)** Distribution by metabolic cell death mechanism: autophagy-dependent cell death (29,142), ferroptosis (28,529), apoptosis (7,626), cuproptosis (2,404), lysosome-dependent cell death (707), alkaliptosis (706), necroptosis (617), pyroptosis (381), parthanatos (198), oxeiptosis (132), and entotic cell death (94).

By metabolic category, signatures were distributed: carbohydrate metabolism, 57,563 (23.8%); lipid metabolism, 56,870 (23.5%); amino-acid metabolism, 53,422 (22.1%); metabolism of cofactors and vitamins, 33,485 (13.8%); metabolism of other amino acids, 14,186 (5.8%); nucleotide metabolism, 13,831 (5.7%); and energy metabolism, 12,058 (5.0%) (Figure 2A).

By omic layer, signatures were distributed: miRNA expression, 102,272 (42.4%); transcript-isoform expression, 65,590 (27.2%); gene expression, 31,694 (13.1%); CpG methylation, 25,400 (10.5%); CNV, 8,608 (3.6%); somatic mutation, 7,827 (3.2%); and protein expression, 24 (<0.1%) (Figure 2B).

Over 70,000 signatures were mapped to metabolic cell death mechanisms, primarily autophagy-dependent cell death, 29,142 (41.3%), and ferroptosis, 28,529 (40.4%), followed by apoptosis, 7,626 (10.8%); the remaining mechanisms were less frequent (Figure 2C).

Despite substantial variation in the absolute number of signatures across tumor types, the relative proportions of signatures across omic layers, metabolic categories, and metabolic cell death mechanisms remain highly consistent across tumor types, as shown in the right panels of Figure 2. This proportional stability indicates that the observed distribution is unlikely to be driven solely by differences in feature abundance or sample size, but instead reflects a conserved structural organization of metabolic regulatory programs across cancers. To confirm this, pairwise Pearson correlations of compositional vectors across the 33 tumor types revealed near-perfect concordance of proportions across metabolic categories (median r = 0.996; IQR: 0.993–0.998), metabolic cell death mechanisms (median r = 0.991; IQR: 0.975–0.997), and omic layers (median r = 0.959; IQR: 0.915–0.980). Furthermore, Cramér’s V effect sizes were minimal (<0.11 across all dimensions), indicating a very weak association between distributional profiles and cancer type, further supporting their structural conservation.

### 3.5 Signatures association with tumor phenotype, clinical outcome, and tumor microenvironment profile

Having resolved coherent metabolic states, we next examined whether these signatures share upstream regulatory control. Signatures showed significant associations with MSI, TMB, and, most prominently, TSM across all omic layers, metabolic categories, and metabolic cell death mechanisms (Supplementary Figures S1; Supplementary Tables S11-S13). Most associations corresponded to dual phenotypic profiles, followed by pro-tumoral and anti-tumoral signatures (Supplementary Figure S2; Supplementary Tables S14-S16). When examining immune phenotypes, cold-associated signatures predominated across datasets, whereas hot and variable immune profiles represented smaller fractions (Supplementary Figure S3; Supplementary Tables S17-S19).

A comprehensive summary of counts and percentages for each omic layer, metabolic category, and metabolic cell death mechanism is provided in Supplementary Tables S17-S19, while the complete dataset of individual associations for all signatures and tumor types is available in Dataset S3.

Regarding the clinical endpoints, the prognostic associations—capturing both risk-enhancing and protective effects—are presented in Supplementary Tables S20-S31 and summarized in Supplementary Figures S4-S7.

### 3.6 Signature nomenclature

**FIGURE 3:**
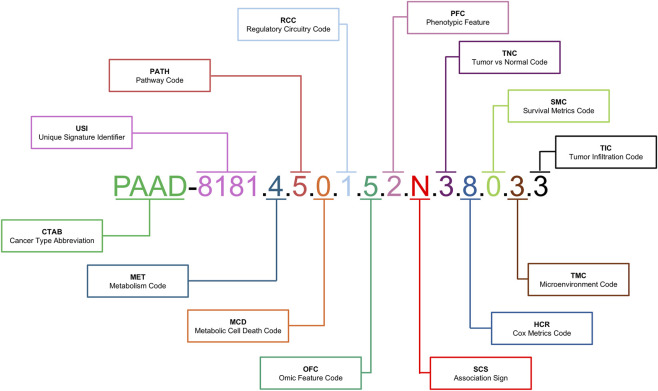
Hierarchical nomenclature system of omic-specific metabolic signatures in OncoMetabolismGPS. This figure illustrates the extended nomenclature and coding structure used to uniquely identify and rank multi-omic metabolic signatures. Each signature is encoded by a 14-token alphanumeric string that integrates tumor context, metabolic and regulatory dimensions, phenotypic behavior, and immune classification. The coding sequence follows the format: [CTAB]-[USI].[MET].[PATH].[MCD].[RCC].[OFC].[PFC].[SCS].[TNC].[HRC].[SMC].[TMC].[TIC]. Here, CTAB is the TCGA tumor abbreviation (cancer abbreviations defined in Supplementary Table S7), USI the unique series identifier, MET the metabolic category (e.g., lipid, amino acid, carbohydrate), PATH the pathway index within that category, MCD the metabolic cell death flag, RCC the regulatory circuitry code flag for interaction(s) with common regulators, OFC and PFC the omic and phenotypic layer codes, SCS the correlation sign (positive or negative), TNC the tumor-versus-normal expression code, HRC and SMC the hazard-direction and survival worst-group templates across the survival metrics DSS (disease-specific survival), DFI (disease-free interval), PFI (progression-free interval), and OS (overall survival), TMC the tumor-microenvironment class (anti-tumoral, dual, or pro-tumoral), and TIC the immune phenotype (hot, cold, or variable). Relative to the earlier nomenclature used for RCD-associated signatures (Li Q. et al., 2024), three new fields are introduced: MET (metabolism code), PATH (pathway code), and RCC (regulatory circuitry code), extending the system from 11 to 14 components.

To enable unambiguous identification and cross-referencing of all signatures, we implemented a structured nomenclature system adapted from our previously published framework for RCD-associated signatures (Rodrigues de Souza et al., 2025) and extended for the metabolic context of the present study. The original RCD system contained eleven hierarchical tokens encoding tumor, omic, phenotypic, prognostic, and immune attributes. In the OncoMetabolismGPS framework, this structure was expanded to a 14-component code that now also captures the metabolic and regulatory dimensions specific to this framework (Figure 3; Supplementary Material - Method C).

Each signature identifier follows the general token format [CTAB]-[USI].[MET].[PATH].[MCD].[RCC].[OFC].[PFC].[SCS].[TNC].[HRC].[SMC].[TMC].[TIC]. To prevent any ambiguity, it is important to emphasize that this bracketed sequence is not a placeholder for missing data, but rather the formalized algebraic schema that encodes, in compact form, its tumor type, metabolic category, omic layer, phenotype association, survival behavior, and immune classification (Supplementary Material - Method C). Three new fields were introduced relative to the prior nomenclature:

[MET] (Metabolism code) shows the metabolic domain (e.g., lipid, amino acid, carbohydrate).
[PATH] (Pathway code) denotes the KEGG pathway index within each metabolic domain.
[RCC] (regulatory circuitry code) identifies whether the signature takes part in a convergent or divergent regulatory pair within its tumor context.

Together, these tokens provide a lossless, highly structured, and machine-readable representation of each signature’s biological context and analytical metadata. While optimized for computational parsing, the nomenclature is fully decipherable using the hierarchical logic illustrated in Figure 3, with complete token definitions and codebooks available in Supplementary Material - Method C.

Because the signature compendium (Dataset S3) includes both single- and multi-element configurations, and because individual metabolic components may take part in different biological contexts, the same molecular elements can appear in distinct signatures across tumor types whenever they satisfy layer-specific, phenotypic, prognostic, and immune-context criteria. The *AKR1C1* + *AKR1C2* pair illustrates this principle: these genes formed tumor-specific signatures in GBM, KIRC, KIRP, LUAD, PAAD, PRAD, SKCM, THYM, and UCS (abbreviations in Supplementary Table S7), each reflecting coherent associations with TSM and a cold immune microenvironment at both the gene-expression and DNA-methylation layers. In prostate adenocarcinoma (PRAD), for example, the PRAD-specific signature (PRAD-1119.4.13.6.0.6.3.N.2.5.0.1.3) consistently mapped to a cold immune microenvironment and exhibited an adverse prognostic profile, with significant effects in overall survival (p = 0.0136) and disease-specific survival (p = 0.00318). This case shows how individual metabolic genes can give rise to distinct, cancer-type-specific metabolic signatures, each shaped by its surrounding phenotypic, clinical, and immune context.

### 3.7 Shared molecular regulatory interactions within signatures

To bridge metabolic signatures with their upstream regulatory circuitries, we examined whether the components of each signature were connected through shared molecular regulators. For enzyme-coding gene signatures, we intersected experimentally validated miRNA–mRNA and lncRNA–mRNA interactions, keeping only those regulators common to all members. For miRNA- and lncRNA-based signatures, we applied the equivalent criterion in the reverse regulatory direction, identifying coding genes that were co-targeted by all non-coding members.

Of the 241,415 total signatures, 57,033 (23.6%) were multi-member, whereas the remaining 184,382 (76.4%) comprised single elements. All percentages below are expressed relative to the total number of signatures. Among the multi-member signatures, 11,021 (4.6%) were linked by at least one shared regulator, whereas 46,012 (19.1%) showed no common regulatory connection among their members. Among the single-member signatures, 171,084 (70.9%) were connected to at least one common upstream regulator, while 13,298 (5.5%) had no known regulatory connection. Thus, in total, 182,105 signatures (75.4%) exhibited at least one molecular regulatory interaction across cancer types.

As an illustrative example, the metabolic signature PAAD-1340.5.4.0.1.6.3.N.3.3.0.3.3—composed of the genes *NNT* and *SIRT4*, both annotated to nicotinate and nicotinamide metabolism within the cofactors and vitamins category—shared a regulatory module of five experimentally validated upstream microRNAs: hsa-miR-103a-3p, hsa-miR-15a-5p, hsa-miR-15b-5p, hsa-miR-16-5p, and hsa-miR-195-5p. The presence of this multi-miRNA set illustrates how multiple metabolic target genes can converge under the control of the same post-transcriptional regulators, highlighting the integrative architecture whereby functionally coordinated metabolic units arise from the intersection of shared biochemical relatedness and common regulatory control.

### 3.8 Meaningful convergent and divergent regulatory relationships between shared regulators and signatures

This section makes up the core output of the framework: a multi-omic atlas of convergent and divergent metabolic regulatory circuitries reconstructed across 33 cancer types. To characterize the directionality of regulatory influence, we classified each regulator–signature pair as either convergent or divergent based on the sign of their association across molecular, phenotypic, and clinical dimensions. A convergent relationship occurs when the regulator and its target signature exhibit correlated behavior, acting in the same biological direction across phenotypic and prognostic contexts. Conversely, a divergent relationship reflects opposite association directions, implying regulatory compensation or feedback within the metabolic circuitry.

For signatures with shared regulators, we compared the direction of association between each signature and its regulator across tumor phenotypes (MSI, TMB, TSM), survival metric outcomes (overall survival (OS), disease-specific survival (DSS), disease-free interval (DFI), and progression-free interval (PFI)), and immune-infiltration profiles (hot, cold, variable).

This analysis evaluated the subset of signatures engaged in measurable regulatory interactions across phenotypic, prognostic, and immune dimensions. In total, 84,270 unique signatures met these criteria and exhibited at least one evaluable molecular regulatory interaction. Because a single signature can be linked to multiple regulators, and individual regulators can interact with several signatures, the total number of regulator–signature relationships analyzed for convergence and divergence exceeded the number of unique signatures, totaling 204,591 distinct interactions (Dataset S4).

Across all regulatory interactions, 27.2% (55,759) displayed only divergent associations, while 24.4% (49,856) exhibited only convergent associations. To evaluate domain-specific influence, we quantified how frequently each biological domain demonstrably steered the final interaction consensus (as defined in the Final concordance summary variable in Dataset S4). Among these dimension-specific evaluations, interactions demonstrably influenced by the immune profile accounted for the largest proportion (48.4%, 98,976), followed by those influenced by tumor phenotypes (34.2%, 70,072) and generic Cox regression endpoints (29.3%, 59,980). Notably, when evaluating explicit Overall Survival (OS) outcomes, precisely 21.0% (43,046) of interactions exhibited a strictly convergent or divergent directionality, explicitly excluding associations with a mixed survival impact.

**FIGURE 4:**
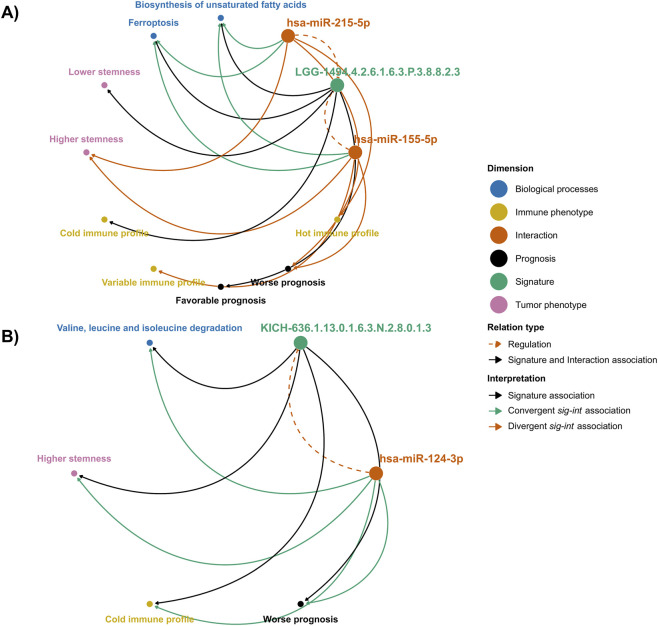
Convergent and divergent regulatory circuitries across tumor contexts. **(A)** In LGG, a lipid-metabolism signature composed of *FADS1* and *SCD*—annotated to the biosynthesis of unsaturated fatty acids and ferroptosis—formed the metabolic signature LGG-1494.4.2.6.1.6.3.P.3.8.8.2.3, which displayed a divergent regulatory circuitry relative to its paired regulatory interactions (comprising miR-215-5p and miR-155-5p). The metabolic signature was associated with higher TSM, a cold immune microenvironment, and a favorable prognosis, whereas the regulatory signature showed the opposite pattern—lower TSM, hot immune profiles, and poor prognosis. This antagonistic configuration illustrates metabolic–immune trade-offs, in which metabolic advantage and immune visibility are regulated in opposing directions within the same tumor context. **(B)** In KICH, a metabolic signature composed of *ACAT2* and *HADH*, linked to branched-chain amino-acid degradation, tryptophan metabolism, and the butanoate pathway, formed the metabolic signature KICH-636.1.13.0.1.6.3.N.2.8.0.1.3 and exhibited convergent regulation with its paired regulatory interaction (defined by miR-124-3p). Both signatures showed negative correlations with TSM, association with cold immune profiles, and unfavorable prognostic behavior, demonstrating alignment across molecular, phenotypic, and clinical dimensions. Together, panels **(A)** and **(B)** illustrate how divergent circuitries mark axes of adaptive metabolic–immune plasticity, whereas convergent circuitries stabilize coordinated metabolic programs within tumor contexts.

In brain lower grade glioma (LGG), a lipid-metabolism signature composed of *FADS1* and *SCD*—annotated to unsaturated fatty acid biosynthesis and the ferroptosis pathway—exhibited a divergent regulatory pattern. The metabolic signature LGG-1494.4.2.6.1.6.3.P.3.8.8.2.3 showed a positive association with TSM at the gene-expression level (*ρ* = 0.216), corresponded to a cold immune microenvironment, and was associated with a protective prognostic effect in Cox analyses. In contrast, its shared upstream regulators, hsa-miR-215-5p and hsa-miR-155-5p, displayed opposite trends, including negative correlations with TSM (*ρ* = −0.529 and −0.253, respectively), associations with a hot immune phenotype, and a risky prognostic profile (Figure 4A; Dataset S4). Together, the metabolic signature and its shared upstream regulators exemplify a divergent metabolic regulatory circuitry in which the regulatory layer acts in an opposing biological direction relative to the metabolic signature.

In KICH, a metabolic signature composed of *ACAT2* and *HADH*—annotated to branched-chain amino acid degradation pathway—exhibited a convergent regulatory pattern. The metabolic signature KICH-636.1.13.0.1.6.3.N.2.8.0.1.3 showed a negative association with TSM (*ρ* = −0.52), corresponded to a cold immune microenvironment, and was linked to a risky prognostic effect in Cox analyses. Its shared regulator, hsa-miR-124-3p displayed the same directional behavior, including a negative correlation with TSM (*ρ* = −0.45), a cold immune phenotype, and an adverse prognostic profile. This coherence across molecular, phenotypic, immune, and clinical dimensions defines a convergent metabolic regulatory circuitry, in which both the metabolic and regulatory signatures act in the same biological direction within the tumor context (Figure 4B; Dataset S4).

Together, these results define a landscape of context-dependent regulatory interactions across human cancers, establishing the foundation for identifying multi-omic metabolic circuitries composed of paired signatures that interact functionally within the same tumor context.

### 3.9 Multi-omic metabolic regulatory circuitries

In our analytical framework, a metabolic regulatory circuitry is operationally defined as a cancer type-specific pair of omic signatures in which one signature comprises enzyme-coding genes involved in metabolic pathways and the other comprises non-coding RNA regulators (miRNAs or lncRNAs) with experimentally validated interactions with those genes. For each pair, all components of the regulatory signature are linked to all components of the target signature through validated molecular interactions, ensuring complete cross-connectivity. These paired signatures were analyzed for directionality of association across molecular, phenotypic, and clinical dimensions to determine whether their regulatory relationship was convergent, when both signatures exhibited aligned associations with tumor features (e.g., both linked to risk or both to protection), or divergent, when their associations were inverse (e.g., one enriched in protective phenotypes while the other corresponded to risk). Circuitries identified in this manner represent structured regulatory modules coupling metabolic gene programs to their non-coding regulators within defined tumor contexts.

From the 204,591 distinct regulator–signature interactions identified in Dataset S4, we generated composite interaction signatures and compared them against the catalog of omic-specific metabolic signatures defined previously (Dataset S3) to determine overlaps. This mapping revealed 24,796 matching pairs between the two datasets (Dataset S5).

Exploratory analysis of the signature–interaction pairs revealed that the vast majority (23,288 pairs; 93.9%) were classified as unique in both datasets, showing that each signature and its corresponding interaction involved a single, specific molecular element. A smaller subset (904 pairs; 3.6%) comprised unique signatures linked to multiple interactions, while 568 pairs (2.3%) exhibited the opposite pattern—multiple components within the signature but a single upstream regulator in the interaction. Only 36 pairs (0.14%) contained multiple components in both the signature and the interaction, representing multifactorial regulatory associations.

**FIGURE 5:**
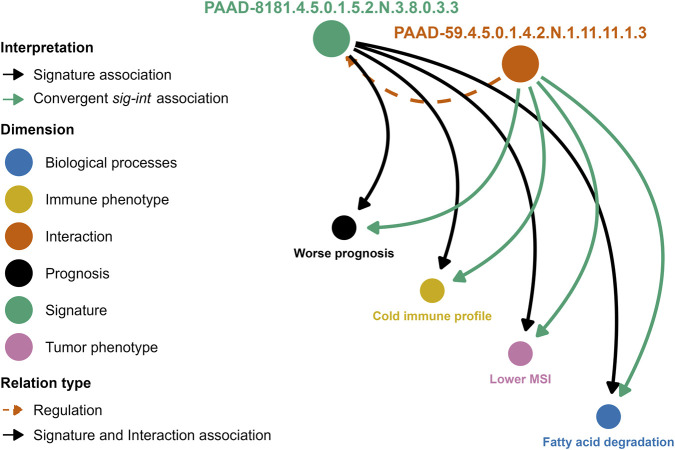
Convergent metabolic regulatory circuitry in PAAD. In PAAD, a multi-element transcriptomic signature PAAD-8181.4.5.0.0.5.2.N.3.8.0.3.3—composed of *ALDH7A1* transcripts ENST00000503281 and ENST00000553117 and annotated to the fatty-acid degradation pathway—forms a convergent circuitry with the paired regulatory signature PAAD-59.4.5.0.1.4.2.N.1.11.11.1.3, comprising hsa-let-7b-5p and hsa-miR-193b-3p. Both signatures showed negative associations with MSI (*ρ* = −0.46 and −0.38) and displayed coherent high-risk prognostic profiles across OS, DSS, and DFI survival metrics. Immunologically, each signature corresponded to a pro-tumoral, cold microenvironment, indicating reduced immune infiltration. This alignment across molecular, phenotypic, survival, and immune dimensions exemplifies a convergent metabolic regulatory circuitry in which metabolic and regulatory signatures act in the same biological direction within the tumor context.

To illustrate a representative example, we selected a pair containing multiple targets in the signature and regulators in the interaction, representing convergent associations (Figure 5). The signature PAAD-8181.4.5.0.0.5.2.N.3.8.0.3.3, specific to the fatty acid degradation pathway, is composed of the transcripts ENST00000503281 and ENST00000553117 of the *ALDH7A1* (Aldehyde Dehydrogenase 7 Family Member A1) gene and functionally integrates with the miRNA interaction hsa-let-7b-5p + hsa-miR-193b-3p (PAAD-59.4.5.0.1.4.2.N.1.11.11.1.3). In PAAD patients, this association is characterized by a convergent relationship between transcript and miRNA expression layers, both correlated with the MSI phenotype. The negative correlations observed for the signature (*ρ* = −0.46; padj = 0.0064) and for the interaction (*ρ* = −0.38; padj = 5.3 × 10^−6^) show a coordinated regulatory effect. In survival analyses, both the signature and the interaction exhibited a high-risk profile in a convergent manner across DSS, DFI, and OS survival metrics, demonstrating prognostic coherence between the omic layers. For the transcriptomic signature, p-values were 0.0264 (OS), 0.0293 (DSS), and 0.0198 (DFI), showing significant associations with worse survival outcomes. For the miRNA interaction, the associations were even stronger, with p = 8.13 × 10^−5^ (OS), p = 5.15 × 10^−4^ (DSS), and p = 4.07 × 10^−4^ (DFI). From an immunological and microenvironmental perspective, both layers displayed concordant profiles, being associated with a pro-tumoral and immunologically cold microenvironment, consistent with reduced immune infiltration and unfavorable clinical outcome.

### 3.10 OncoMetabolismGPS Shiny application for interactive exploration

To facilitate access, navigation, and interpretation of the multidimensional metabolic signatures generated in this study, we developed OncoMetabolismGPS, an interactive Shiny-based web application (https://oncometabolismgps.be/). The acronym OncoMetabolismGPS reflects both the structural logic and navigational purpose of the framework. Analogous to a guided positioning system, the platform enables systematic identification and contextualization of omic-specific metabolic signatures based on their molecular composition and associated phenotypic, immune, and clinical attributes. The structured 14-token nomenclature functions as a unique identifier that encodes the biological context of each signature, allowing precise retrieval, comparison, and cross-referencing across tumor types.

Rather than representing a spatial model, the framework operates as an organized reference system in which signatures, pathways, and regulatory circuitries can be explored through their encoded attributes. This design supports efficient navigation across multiple layers of biological information and facilitates the interpretation of complex regulatory relationships derived from large-scale datasets.

The OncoMetabolismGPS Shiny application operationalizes this principle by providing an interactive interface that allows users to explore the organization of metabolic signatures in cancer. Through layered visualization modules, users can traverse from single enzyme-coding genes or non-coding regulators to higher-order metabolic signatures and their convergent or divergent regulatory circuitries, examining how molecular patterns align with phenotypic, prognostic, and immune dimensions across tumor contexts. The interface integrates all omic-specific metabolic signatures with their associated metabolic pathways, RCD annotations, tumor phenotypes (MSI, TMB, TSM), survival outcomes (OS, DSS, DFI, PFI), immune infiltration profiles, and shared regulatory circuitries.

Users can query signatures by cancer type, omic layer, metabolic pathway, cell-death mechanism, phenotypic association, immune-microenvironment class, or specific gene, miRNA, or lncRNA identifiers. For each selected signature, the interface displays its molecular components, the regulatory interactions defining convergent or divergent circuitries, association statistics across phenotypic and clinical dimensions, immune-context classification, and downloadable data tables and visual summaries. The application provides an operational framework for investigating how metabolic reprogramming shapes tumor behavior across biological contexts and serves as a resource for hypothesis generation, pathway prioritization, identification of metabolic vulnerabilities, and selection of biomarker candidates for translational validation. All custom R scripts used for preprocessing, association testing, signature construction, regulatory analyses, and circuitry derivation are publicly available in the associated GitHub repository.

## 4 Discussion

This study presents a systematic strategy for identifying and organizing multi-omic regulatory associations across cancer types. The analytical strategy emphasizes the detection of consistent patterns across molecular, phenotypic, immune, and clinical dimensions, enabling the construction of biologically coherent metabolic regulatory circuitries.

Within this context, the results should be interpreted as a structured, hypothesis-generating resource that prioritizes internal consistency and cross-layer concordance, providing a foundation for downstream experimental validation and targeted functional studies.

### 4.1 Integrated view of metabolic reprogramming and cell-fate control

By integrating genomic, epigenomic, transcriptomic, immune, and clinical information across tumor types, this study provides a systems-level framework for interpreting how metabolic regulatory architectures shape tumor behavior, immune context, and patient-relevant phenotypes in cancer.

Metabolic rewiring, immune modulation, and RCD constitute interdependent axes that collectively dictate tumor behavior (Faubert et al., 2020; Mao et al., 2024; Dalseno et al., 2025). Metabolic programs intersect with apoptotic, ferroptotic, autophagic, and necrotic pathways, influencing cell-fate decisions in a context-dependent manner. These same metabolic alterations promote immune evasion through nutrient competition, redox remodeling, and disrupted antigen presentation, while microenvironmental stressors—hypoxia, ROS accumulation, and amino-acid limitation—activate specific RCD modules that feed back into tumor evolution. This perspective highlights the need for frameworks capable of capturing metabolic regulation as an integrated architecture rather than a collection of isolated associations.

The metabolic–immune–RCD axis is further shaped by local environmental constraints such as pH, nutrient gradients, and stromal interactions, as illustrated in injury models where metabolic stress elicits distinct RCD patterns depending on substrate availability (Faubert et al., 2020; Mao et al., 2024; Dalseno et al., 2025). Within this integrated landscape, tumor metabolism functions as a central regulatory hub that links survival, death, and immune escape programs. This systems-level view motivated the development of our multi-omic framework designed to reconstruct metabolic signatures and their governing regulatory logic across diverse cancers.

The integration between cellular metabolism and RCD mechanisms shows that metabolic pathways do not act in isolation. They form a multifunctional system in which metabolic enzymes take part in various physiological and adaptive processes, conferring plasticity to cellular responses (Tang et al., 2021). The regulation of these mechanisms depends strongly on the metabolic state. Processes such as ferroptosis and autophagy respond to fluctuations in nutrient supply, oxidative stress, and the performance of cellular bioenergetic pathways. For example, in cases of acute kidney injury, the energetic metabolism of tubular epithelial cells is reprogrammed, involving coordinated changes in lipid, glucose, and amino acid metabolism. This reprogramming is associated with the activation of different RCD forms, establishing a metabolism–cell death axis that influences both tissue progression and repair (Zhao et al., 2023). Similarly, in colorectal carcinomas (rectum adenocarcinoma (COAD) ad rectum adenocarcinoma (READ)), oncogene-driven metabolic reprogramming alters glycolysis, glutaminolysis, one-carbon metabolism, and lipid pathways. These changes support cell proliferation, immune evasion, and therapeutic resistance, while the tumor microenvironment both modulates and is modulated by these routes (Nenkov et al., 2021).

Alternative forms of metabolic cell death, such as cuproptosis, reinforce this interdependence: therapies that redirect tumor metabolism from a glycolytic to a more oxidative state increase sensitivity to copper-induced toxicity, intensifying cell death (Zhang et al., 2024).

Taken together, these observations indicate that the metabolic state is a key determinant of cellular vulnerabilities and fate. Based on this, the study proposes a multidimensional Pan-Cancer framework that integrates metabolic, phenotypic, and clinical information to identify signatures and molecular interactions within a regulatory circuitry. Implemented in the OncoMetabolismGPS application, this approach supports a model in which tumor metabolism exhibits a layered regulatory architecture linking biochemical activity, RCD mechanisms, tumor aggressiveness, and immune response. This approach enables the identification of context-dependent metabolic vulnerabilities that may serve as strategic points for future basic and applied investigations with potential therapeutic relevance.

### 4.2 A pan-cancer landscape of multi-omic metabolic signatures

Across 33 tumor types, we constructed a unified atlas of metabolic signatures integrating multi-omic, phenotypic, immunological, and prognostic information. These signatures define coherent metabolic states based on pathway membership, phenotypic directionality, and clinical consistency, with broad representation across lipid, carbohydrate, amino-acid, and cofactor/vitamin metabolism. Notably, more than 70,000 signatures aligned with RCD processes—especially autophagy-dependent cell death and ferroptosis—underscoring the tight coupling between metabolic reprogramming and RCD.

TSM accounted for most significant phenotypic associations, with TMB and MSI contributing secondarily, while immune-cold patterns predominated across metabolic categories. Clinically, signatures segregated into protective and high-risk groups, reflecting divergent metabolic fitness states. Altogether, this atlas offers a structured overview of metabolic behavior across cancers and forms the analytical foundation for reconstructing metabolic regulatory circuitries.

By grouping elements only when they share the same metabolic pathway, RCD mechanism, omic–phenotypic concordance, and immune microenvironment type, over 240,000 metabolic signatures specific to each omic layer were generated, in which multimolecular signatures represent phenotypes coordinated by multiple components, while single-component signatures indicate isolated or context-specific dependencies. Because many metabolic enzymes and regulatory RNAs take part in different pathways and death mechanisms, we allowed the same target to appear in multiple signatures, preserving its pleiotropy. This redundancy is therefore biological, not methodological.

The metabolic signatures showed strong associations with the tumor stem-like phenotype. The recurrent presence of this type of relationship across several omic layers shows that metabolic reprogramming is a defining feature of tumor states with stem-cell-like properties, in which metabolism acts as a central axis of phenotypic plasticity.

For example, serine metabolism reprogramming shows that increased synthesis and utilization favor biosynthetic production, redox control, and chromatin modification, supporting oncogenesis, stemness, and therapeutic resistance (Shunxi et al., 2023). Similarly, inosine-induced reprogramming in engineered immune cells improves mitochondrial function, polyamine synthesis, and epigenetic remodeling toward a stem-like state, increasing persistence and efficacy of CAR-T cells (Klysz et al., 2024). Furthermore, the dependence of several tumor and immune populations on fatty acid β-oxidation (FAO) for survival, maintenance of stemness, metastatic potential, and immune evasion reinforces the central role of metabolic adaptability in determining cell fate under the pressures of the tumor microenvironment (Ma et al., 2018).

T-cell stemness itself is metabolically constrained in this microenvironment, where high levels of extracellular K^+^, lactate, and acidosis modulate the balance between self-renewal and terminal exhaustion, affecting the effectiveness and durability of immunotherapeutic responses (Liu et al., 2023). In summary, tumor metabolism plays a central role in phenotypic plasticity, and stemness emerges as a metabolically sustained cellular condition regulated across multiple omic layers.

### 4.3 Convergent and divergent metabolic regulatory circuitries

A central question emerging from the signature compendium is whether the components within each signature—especially multi-member configurations—are connected by common upstream regulatory elements. To address this, we systematically intersected experimentally validated miRNA–mRNA and lncRNA–mRNA interactions across all components of every signature. For enzyme-gene signatures, only regulators that interact with all signature members were retained; for miRNA- and lncRNA-based signatures, the criterion was reversed, identifying coding genes co-regulated by all non-coding elements. This approach ensures that every retained regulator reflects a complete cross-connectivity pattern within the signature rather than partial or incidental interactions.

This analysis revealed extensive regulatory integration across cancer types: over 75% of all metabolic signatures exhibited at least one shared regulatory interaction, providing a structural substrate for reconstructing convergent and divergent regulatory circuitries. Multi-element signatures frequently mapped to multiple shared regulators, indicating points of coordinated metabolic control, whereas single-element signatures often carried broad regulatory connectivity reflecting the pleiotropy of individual metabolic genes and ncRNAs.

Together, these findings support the interpretation that metabolic signatures are not merely co-annotated metabolic units but regulatory modules anchored in shared ncRNA control, thereby enabling the definition of higher-order regulatory circuitries.

Another important finding was the predominance of cold immune phenotypes across different tumor types, reinforcing the idea that metabolic reprogramming is closely linked to immune cell suppression and exclusion. This pattern is consistent with evidence in non–small cell lung cancer, where metabolic alterations and suppressive stromal networks sustain inactive immune microenvironments and promote resistance to checkpoint blockade therapy (Yu et al., 2025). Similarly, in breast cancer (BRCA), MIF-mediated glycolysis not only stimulates tumor growth and dissemination but also inhibits cytotoxic immunity; its inhibition restores pro-inflammatory immune infiltration and increases the effectiveness of checkpoint inhibitors (Yan et al., 2024). In addition, studies show that early immunosurveillance itself can direct the metabolic reprogramming of tumor cells through non-canonical IFNγ–STAT3 signaling, shaping bioenergetic states that allow cancer cells to compete with infiltrating lymphocytes and maintain an immunosuppressive environment (Tsai et al., 2023). Taken together, these observations indicate that most signatures classified as indicative of cold immune profiles reflect immune-evasion strategies dependent on tumor metabolism.

A representative example is the lipid metabolism–related signature composed of the genes *AKR1C1* and *AKR1C2*, involved in steroid hormone biosynthesis and the ferroptosis pathway. This signature showed concordance across different omic layers, as its association with the stemness phenotype was observed in both gene expression and CpG methylation profiles, encompassing multiple tumor types, including GBM, KIRC, KIRP, LUAD, PAAD, PRAD, SKCM, THYM, and UCS. In all these contexts, it maintained a consistent correlation with cold immune microenvironments, reinforcing the notion that metabolic alterations associated with stemness often coincide with suppressed or desert immune profiles.

In PRAD, the signature involving *AKR1C1* and *AKR1C2* (PRAD-1119.4.13.6.0.6.3.N.2.5.0.1.3) showed an adverse prognostic effect, evidenced by Cox regression analyses for overall survival (p = 0.0136) and disease-specific survival (p = 0.00318). This result is consistent with the known role of AKR enzymes in androgen metabolism. AKR1C2, for example, converts dihydrotestosterone (DHT) into its less active metabolite, 3α-diol; reduced activity of this enzyme in PRAD leads to excessive retention of DHT and favors tumor progression. Pharmacologic inhibition of AKR1C2 has been reported to suppress tumor growth in both *in vitro* and *in vivo* models (Nie et al., 2024). In parallel, AKR1C3 enhances androgen biosynthesis, promoting castration resistance and establishing itself as a validated therapeutic target (Byrns et al., 2011; Adeniji et al., 2013). AKR1C1 also plays an important role in hormonal metabolism, particularly in regulating progesterone homeostasis, and is highly expressed in various hormone-dependent neoplasms. Recent structural and biochemical studies have confirmed the feasibility of developing selective AKR1C1 inhibitors—including synthetic and natural compounds—based on differences in functional domains, co-crystallized binding sites, and polymorphic variants that modulate enzymatic activity and disease susceptibility (Chu et al., 2022).

Thus, the recurrence of the *AKR1C1*+*AKR1C2* signature across different tumor types, its association with transcriptional and epigenetic states related to stemness, and its consistent relationship with cold immune microenvironments illustrate how metabolism, particularly lipid metabolism, simultaneously shapes tumor characteristics and immune-evasion mechanisms. This example highlights the value of representing metabolic dependencies as modular, context-specific signatures. This representation more accurately reflects interactions between metabolism, tumor phenotype, and immune response than one-dimensional approaches.

Among the 241,415 metabolic signatures constructed, 23.6% contained multiple components, whereas 76.4% comprised a single target. In the group of multimolecular signatures, 19.3% shared at least one common regulator, although most did not. In contrast, among single-component signatures, 92.8% were associated with at least one regulator, and only a small fraction had none.

Based on this analysis, we sought to understand the regulatory relationships between signatures of upstream regulators and their metabolic targets that may exhibit convergent or divergent patterns across different biological layers within the same tumor type.

Among 204,591 regulator–signature interactions, divergent patterns predominated (27.2%) compared with convergent ones (24.4%), although the latter formed a recurrent and structurally consistent subset of regulatory circuitries. The immune infiltration domain showed the highest proportion of significant interactions (48.4%), indicating that the immune context represents the main axis of regulatory reconfiguration between signatures and their controllers. Because a single regulator can exert pleiotropic action on several target genes in distinct metabolic pathways, a signature may exhibit both convergent and divergent interactions. This means that the observed effects may result not only from the direct interaction with the signature but also from indirect influences on other targets that differentially modulate the immune, phenotypic, or prognostic landscape of the tumor.

Beyond the predominance of divergent regulatory relationships, the atlas revealed additional quantitative regularities that characterize the multi-omic organization of tumor metabolism. Across all association tests, TSM emerged as the dominant phenotypic axis, accounting for 72.1% of significant associations, whereas TMB and MSI contributed substantially smaller fractions. In the immune domain, cold phenotypes overwhelmingly predominated (81.6%), with hot and variable states representing only minor components of the regulatory landscape. At the molecular level, transcript-isoform expression accounted for 51.7% of all significant associations—outweighing gene expression, methylation, mutation, miRNA, and CNV layers combined. Collectively, these patterns indicate that transcript-level variation, stemness-linked phenotypic shifts, and immunologically cold microenvironments constitute the major axes along which metabolic regulatory circuitries reconfigure across cancer types, complementing the predominance of divergent regulatory behavior observed in the interaction analyses.

It is important to note that the relative contribution of each omic layer to the total number of significant associations may be influenced, in part, by differences in feature dimensionality across datasets. In particular, transcript isoform-level data inherently comprise a larger number of measurable features compared to other omic layers, which may increase the probability of detecting statistically significant associations.

Therefore, the observed prominence of isoform-related signatures should be interpreted in the context of this feature-space asymmetry, rather than as a direct measure of biological dominance. Notably, despite this effect, the recurrence of isoform-associated regulatory patterns across multiple tumor contexts supports their biological relevance beyond purely statistical considerations. This highlights a critical departure from the common paradigm that considers bulk RNA at the gene-level expression as the primary source of candidate biomarkers. While gene-level aggregation is informative, our framework dissects these significant associations to the isoform-specific level, revealing that metabolic reprogramming is frequently driven by a few specific isoforms rather than global gene upregulation. This demonstrates that alternative splicing and isoform switching are the actual drivers of these phenotypic associations, a finding that directly aligns with our recent work establishing that specific transcript isoforms act as the leading drivers of associations in regulated cell death across cancers (Rodrigues de Souza et al., 2025). This level of resolution represents a significant advancement over standard gene-level studies that inherently mask these nuanced regulatory dynamics.

An example of a complex regulatory pattern was observed in the microRNA-based signature (hsa-miR-512-3p + hsa-miR-520e), identified in LUAD-1716.2.13.0.1.4.1.P.2.3.3.2.3). This signature is associated with carbohydrate metabolism, particularly the pyruvate pathway, and is linked to the *LDHD* gene. While the signature itself showed a positive correlation with TMB (*ρ* = 0.188), *LDHD* exhibited a negative correlation with TMB (*ρ* = −0.180). The opposing directions of these correlations indicate a divergent association between the signature and its regulatory partner. Although both microRNAs belonged to the cold immune class, they maintained convergent immune concordance. Survival analyses, however, showed contrasting results: the signature exhibited a risk effect for PFI, whereas the interaction was protective, generating divergence between Cox regression models and survival analyses. In summary, this signature presented immune convergence but phenotypic, Cox, and survival divergence—reflecting the complexity of regulatory interactions that support phenotypic variability in cancer.

Another example was observed in BLCA, with the transcript-level signature (ENST00000367321 + ENST00000618312) (BLCA-8435.5.5.0.0.5.3.P.3.8.8.1.2), associated with cofactor and vitamin metabolism, particularly the one-carbon pool by folate pathway, which is essential for nucleotide synthesis, methylation, and redox control. The signature showed regulatory interactions with hsa-miR-24-3p and hsa-miR-29b-2-5p, two microRNAs linked to TSM. Despite positive and convergent correlations, the biological implications of each were distinct: miR-24-3p appeared in a hot immune context but showed divergent immune concordance, whereas miR-29b-2-5p, classified as variable, displayed convergent immune concordance. In survival analyses, miR-24-3p showed a convergent risk effect in OS, DSS, and PFI, whereas miR-29b-2-5p exhibited a protective effect, evidencing a decoupling between transcriptional regulation and phenotypic behavior. Thus, while miR-24-3p combined immune divergence with phenotypic and Cox convergence, miR-29b-2-5p presented immune and phenotypic convergence but divergence in Cox and survival outcomes.

The LGG *FADS1*–*SCD* signature illustrates how lipid metabolism can generate divergent regulatory circuitries in which metabolic and regulatory layers operate in opposing biological directions. In this context, the balance between polyunsaturated fatty acid synthesis (FADS1) and monounsaturated fatty acid production (SCD) establishes a redox-dependent metabolic state that modulates ferroptotic susceptibility while shaping immune and clinical outcomes. The opposing behavior of their shared upstream regulators, miR-215-5p and miR-155-5p, further indicates that regulatory inputs can reinforce alternative phenotypic states, coupling immune activation with adverse prognosis while the metabolic layer remains associated with a protective profile. This configuration exemplifies how metabolic programs and their regulatory controllers can become decoupled across biological dimensions, revealing context-dependent trade-offs between tumor metabolism, immune engagement, and disease progression.

From a functional perspective, *FADS1* and *SCD* exert complementary yet antagonistic roles in regulating lipid metabolism. FADS1 catalyzes the synthesis of polyunsaturated fatty acids, promoting lipid peroxidation and increasing susceptibility to ferroptosis. In triple-negative BRCA, for example, coexpression of *FADS1* and *FADS2* is associated with poor prognosis, and their pharmacological inhibition reduces both peroxidation and ferroptotic cell death (Lorito et al., 2024). In COAD/READ, FADS1 takes part in an axis that stimulates tumor growth (Xu et al., 2023). Furthermore, the transcription factor Zeb1 activates FADS1 and lipid cofactors such as ACSL4 and ELOVL5, linking mesenchymal plasticity to a lipid-dependent ferroptotic program (Schwab et al., 2024).

On the other hand, SCD converts PUFAs into monounsaturated fatty acids (MUFAs), reducing lipid peroxidation and conferring resistance to ferroptosis. In stomach adenocarcinoma (STAD), SCD is stabilized by USP7-mediated deubiquitination, and its inhibition by the compound DHPO restores ferroptotic sensitivity (Guan et al., 2024). In triple-negative BRCA, overexpression of SCD1/2 maintains resistance to ferroptosis, requiring simultaneous inhibition of FADS1/FADS2 for reversal(Lorito et al., 2024). In pancreatic adenocarcinoma (PAAD), SCD sustains cell survival under lipid restriction, and its inhibition reduces tumor viability (Lien et al., 2021). Interestingly, Zeb1-mediated repression of SCD restores vulnerability to ferroptosis, establishing a functional link between lipid metabolism and cellular plasticity (Schwab et al., 2024).

In the LGG context, the protective effect of the *FADS1*+*SCD* signature, even in the presence of a cold immune microenvironment, suggests that the ratio between PUFAs and MUFAs creates a balanced redox state capable of maintaining a basal ferroptotic vulnerability. While FADS1 promotes ferroptosis sensitivity, SCD acts as a partial compensatory mechanism, preventing excessive oxidative damage. In slow-growing tumors with low inflammation, this metabolic balance may limit both tumor progression and immunopathological damage, in agreement with previous observations regarding these enzymes (Lien et al., 2021; Lorito et al., 2024).

Regarding shared interactions of *FADS1*+*SCD*, in the immune system, both miR-215-5p and miR-155-5p act as potent activators of the immune response, promoting CD8^+^ lymphocyte cytotoxicity through the induction of IFNγ and granzyme B, especially when transferred *via* extracellular vesicles from CD4^+^ T cells (Shin et al., 2022). Their association with a hot immune phenotype but an unfavorable prognosis in LGG indicates a strongly context-dependent effect, possibly related to reactive infiltration in more aggressive tumors, functional differences between tumor and immune cells, and the influence of genomic covariates such as IDH1/2 mutations, 1p/19q co-deletion, and TERT mutations, which reshape the interface between metabolism and immunity. Thus, although their immunostimulatory functions are consistent with the literature (Shin et al., 2022), their clinical impact in LGG is determined by the molecular and cellular landscape of the tumor.

From a therapeutic standpoint, jointly targeting metabolic and immunological pathways represents a promising approach. Inhibition of *SCD* or *FADS1*/*FADS2*, combined with ferroptosis induction, may potentiate immune responses mediated by miR-215-5p and miR-155-5p (Lien et al., 2021; Shin et al., 2022; Guan et al., 2024; Lorito et al., 2024; Schwab et al., 2024). In this way, the *FADS1*+*SCD* signature may act as a biomarker of ferroptosis sensitivity, while specific miRNA profiles may indicate reactive inflammatory states useful for guiding immunotherapy combinations. As evidenced in studies in COAD/READ (Xu et al., 2023), monitoring FADS1-dependent eicosanoid signaling is crucial to prevent pro-tumorigenic side effects, reinforcing the need for contextually calibrated therapeutic approaches that integrate lipid metabolism, ferroptosis, and tumor immunity.

Similarly, the convergent regulatory circuitry identified in KICH—comprising the *ACAT2/HADH* degradation signature and its regulator miR-124-3p—demonstrates how metabolic and regulatory layers can structurally align. The coherent association of this entire circuitry with suppressed stemness, a cold immune microenvironment, and adverse prognostic outcomes exemplifies a specialized, stable metabolic state adapted for immune evasion.

Previous evidence demonstrates that miR-124-3p acts as a tumor suppressor in STAD by directly repressing HRCT1 expression, thereby attenuating ERBB2–MAPK signaling and inhibiting tumor cell proliferation, migration, and invasion (Hou et al., 2023). In KICH, the simultaneous reduction of miR-124-3p and its targets may reflect a compensatory mechanism that limits metabolic stress within an immunosuppressive environment.

The oncogenic enzyme ACAT2 promotes a glycolytic–epigenetic circuit by inducing histone lactylation (H3K18la) and acetylation of the mitochondrial carrier MTCH2, which increases lactate secretion and stimulates M2 macrophage polarization (Yang et al., 2025). The association of *ACAT2*+*HADH* with a cold immune phenotype reinforces the role of *ACAT2* in immunometabolic remodeling and immune evasion. *HADH*, in turn, exhibits a dual and context-dependent behavior: it acts as a tumor suppressor in hepatocellular, gastric, and clear cell renal carcinomas, but as an oncogene in colon cancer and acute myeloid leukemia (Fang et al., 2022; Wang et al., 2022). In renal tumors, reduced *HADH* correlates with AKT pathway activation, PTEN loss, worse clinical outcome, low CD8^+^ lymphocyte infiltration, and increased Tregs. Thus, the combined metabolic repression of *ACAT2* and *HADH* characterizes a mitochondrial–immunosuppressive phenotype in which impaired β-oxidation and altered cholesterol metabolism reshape the tumor immune landscape.

Taken together, these findings reveal an integrated miR-124-3p/*ACAT2*/*HADH* axis that connects epigenetic regulation, energetic metabolism, and immune evasion in KICH. The coordinated reduction of this axis defines tumors metabolically adapted to immunosuppression, suggesting that restoring miR-124-3p expression or rebalancing the *ACAT2*+*HADH* pair may reverse immunometabolic resistance and enhance the response to immunotherapy.

Finally, the multi-omic architecture mapped in PAAD exemplifies how profound metabolic dependencies are structurally cemented by convergent non-coding regulation. By directly coupling *ALDH7A1* transcript variants to the regulatory module of hsa-let-7b-5p and hsa-miR-193b-3p, the tumor establishes an integrated circuitry that strongly correlates with poor prognosis, low microsatellite instability, and an aggressively suppressed, immunologically cold microenvironment.

The gene *ALDH7A1* is highly expressed in pancreatic cancer cell lines and is related to unfavorable outcomes and a strong dependence on fatty acid oxidation (FAO) (Lee et al., 2021). Its function involves detoxifying lipid aldehydes under oxidative stress, generating NADH, and connecting redox balance to energy production. Inhibition of *ALDH7A1* decreases cellular oxygen and ATP consumption, resulting in tumor regression in preclinical models (Lee et al., 2021). The observed pattern of high *ALDH7A1* expression combined with low MSI and a cold immune microenvironment reinforces a model in which the activity of this enzyme creates a redox–energetic state that favors tumor growth and hampers the antitumor immune response.

This behavior is consistent with studies in non–small cell lung cancer and BRCA, which associate *ALDH7A1* with so-called “immune-desert” microenvironments (Stoll et al., 2019). Furthermore, enzymes of the ALDH family, including ALDH7A1, sustain cancer stem cell programs, promoting self-renewal, drug resistance, and tumor progression (Lavudi et al., 2024). *ALDH7A1* overexpression also activates the JAK–STAT and mTOR pathways, contributing to the maintenance of cell growth (Liang et al., 2025). Thus, the association between poor prognosis and suppressed immunity likely reflects the presence of metabolically adapted tumor subpopulations capable of surviving oxidative stress and immune pressure.

From a regulatory perspective, hsa-let-7b-5p is a tumor suppressor miRNA known for restricting proliferation and mitotic escape by targeting genes such as *AURKB, BIRC5, EZH2, PLK1,* and *TK1*, promoting apoptosis and increased cell survival (He et al., 2018; Xi et al., 2019; Zhu et al., 2023; Vats et al., 2025a; Vats et al., 2025b). Loss of its function results in derepression of metabolic and proliferative pathways. hsa-miR-193b-3p shows a comparable pattern, regulating genes involved in fatty-acid oxidation and apoptosis. The functional convergence between let-7b-5p and miR-193b-3p suggests the existence of a cooperative metabolic control module whose dysregulation amplifies oxidative metabolism and reinforces immune exclusion in PAAD.

Taken together, these results describe a metabolic–immunosuppressive circuitry in which ALDH7A1 drives oxidative metabolism and immune evasion, while the loss of let-7b-5p and miR-193b-3p removes the post-transcriptional brakes that would otherwise contain this process. This integration between metabolic reprogramming, immunosuppression, and adverse clinical outcome positions oxidative metabolism as a central element of tumor aggressiveness in pancreatic cancer.

Our results support a model in which tumor metabolism functions as an n-dimensional regulatory space, interdependently integrating genetic, epigenetic, and transcriptomic layers with tumor, immune, and clinical phenotypes. Within this analytical framework, a metabolic regulatory circuitry is defined as a paired relationship between two omic signatures in a specific cancer type: one composed of non-coding RNA regulators and the other formed by metabolically validated targets. Each regulatory element interacts with each target element, forming coherent pairs whose combined behavior determines whether control is convergent or divergent across molecular, phenotypic, and clinical layers. Divergent circuitries reflect the canonical regulatory logic, in which regulator and target exert opposite biological effects—for example, one associated with risk and the other with protection. Conversely, convergent circuitry shows cooperative reinforcement, in which both act in the same direction on metabolism, phenotype, and prognosis. Together, these modules outline the architectures through which non-coding RNAs modulate metabolic state, tumor progression, and the immune context.

### 4.4 Strengths and limitations

The strength of this work derives from its integrative design, conceptual organization, and practical applicability. The study introduces an analytical framework capable of systematically integrating multiple omic layers to reconstruct metabolic regulatory circuitries across tumor types. By organizing associations into omic-specific metabolic signatures linked to phenotypic, immune, and clinical dimensions, the framework enables the identification of coherent biological patterns that extend beyond isolated molecular correlations.

In this context, OncoMetabolismGPS serves as an operational platform that translates these analytical principles into an accessible exploratory resource. Implemented as an interactive Shiny application, the system integrates molecular composition, tumor phenotypes, immune states, and prognostic information within a unified interface. This environment enables dynamic navigation across regulatory circuitries and supports hypothesis generation from large-scale public datasets.

From a translational perspective, the metabolic signatures identified show potential to improve patient stratification, guide targeted therapeutic strategies, and reveal context-dependent metabolic vulnerabilities. The observation that distinct signatures share common regulatory elements suggests that such regulators may act as nodal control points capable of modulating tumor metabolic states associated with aggressiveness, immune exclusion, or susceptibility to regulated cell death. Representative examples, including pathways involving FADS1/FADS2/SCD, USP7–SCD, and enzymes such as HADH and ACAT2, illustrate how the framework integrates molecular and phenotypic information into biologically interpretable regulatory structures (Fang et al., 2022; Shin et al., 2022; Wang et al., 2022; Song et al., 2023; Xu et al., 2023; Guan et al., 2024; Lorito et al., 2024; Yang et al., 2025).

Despite its conceptual and methodological advances, this study presents limitations that must be acknowledged in a balanced manner. The inferred regulatory relationships are based on multi-omic associations aligned across molecular, phenotypic, immune, and clinical domains. While this approach enables the identification of consistent and biologically meaningful patterns, it does not establish direct causal relationships and should be interpreted as a hypothesis-generating framework requiring downstream experimental validation.

The analyses were conducted predominantly using bulk RNA-seq data, which may dilute signals arising from cellular heterogeneity and obscure metabolic states specific to particular cell populations or microenvironmental niches. The incorporation of single-cell and spatial transcriptomics data represents a natural extension of this framework and may further refine the identification of context-specific regulatory circuitries.

Another limitation arises from the reliance on KEGG as the primary source for metabolic pathway annotation. Although KEGG provides a curated and hierarchically structured representation of metabolic processes that supports reproducibility, it may not capture context-specific metabolic rewiring or tumor-type-specific adaptations. Future extensions incorporating complementary resources such as Reactome, MetaCyc, or genome-scale metabolic models may broaden pathway resolution and enhance biological interpretation.

Finally, although the convergence–divergence framework provides a structured approach for integrating metabolism, tumor phenotype, immune context, and clinical outcomes, the translational implications of these relationships remain exploratory and require further validation in experimental and clinical settings.

## 5 Conclusion

This work introduces a unified, multi-omic framework that resolves tumor metabolism into modular, context-specific signatures and their shared regulatory circuitries, linking metabolic pathways to RCD mechanisms, tumor phenotypes (MSI, TMB, TSM), immune profiles (hot/cold/variable), and clinical outcomes across 33 cancers. By systematically integrating enzyme-coding genes, non-coding regulators (miRNAs, lncRNAs), and transcript isoforms, and by enforcing concordance across molecular, phenotypic, immune, and prognostic dimensions, we delineate a layered regulatory architecture in which biochemical activity, cell-fate programs, and microenvironmental pressures co-evolve. The resulting atlas of >240k omic-specific metabolic signatures reveals both conserved and tumor-type-restricted dependencies, with a predominance of immune-cold states and extensive domain-specific convergence/divergence between signatures and their regulators - an organizational principle rarely interrogated in prior studies.

Two mechanistic patterns emerge with translational relevance. First, convergent interactions (e.g., *ACAT2* + *HADH* with miR-124-3p) denote stable disease-driving programs in which metabolic dependency and regulatory control align across molecular, immune, and clinical domains. Second, divergent interactions (e.g., *FADS1* + *SCD* with miR-155-5p/miR-215-5p) expose adaptive fault lines where proliferative/stemness advantages trade off against immune visibility or ferroptosis liability, suggesting entry points for therapeutic sensitization. Together, these patterns argue that metabolic state is not a single hallmark but an adaptive, reconfigurable regulatory landscape whose topology can be read out by signatures and perturbed through shared regulators.

Beyond cataloging, OncoMetabolismGPS operationalizes this framework, enabling hypothesis generation, patient stratification by metabolic state, prioritization of regulators as sensitizers, and rational design of combination strategies that pair metabolic rewiring with cell death-linked interventions and immunotherapy. While causal directionality and cell-type resolution require experimental perturbation and single-cell/spatial validation, the present study establishes the conceptual and analytical foundations for prospective biomarker development and biomarker-guided trials.

## Data Availability

The original contributions presented in the study are publicly available. This data can be found here: https://github.com/BioCancerInformatics/Multi-omic-Oncometabolism-GPS.
