# Supplementary figures and images for: A pan-cancer atlas of metabolic regulatory circuitries integrating multi-omic, immune, and clinical dimensions
Source: Front Mol Biosci. 2026 May 26;13:1845099. doi: 10.3389/fmolb.2026.1845099 (PMC13261907; doi:10.3389/fmolb.2026.1845099)

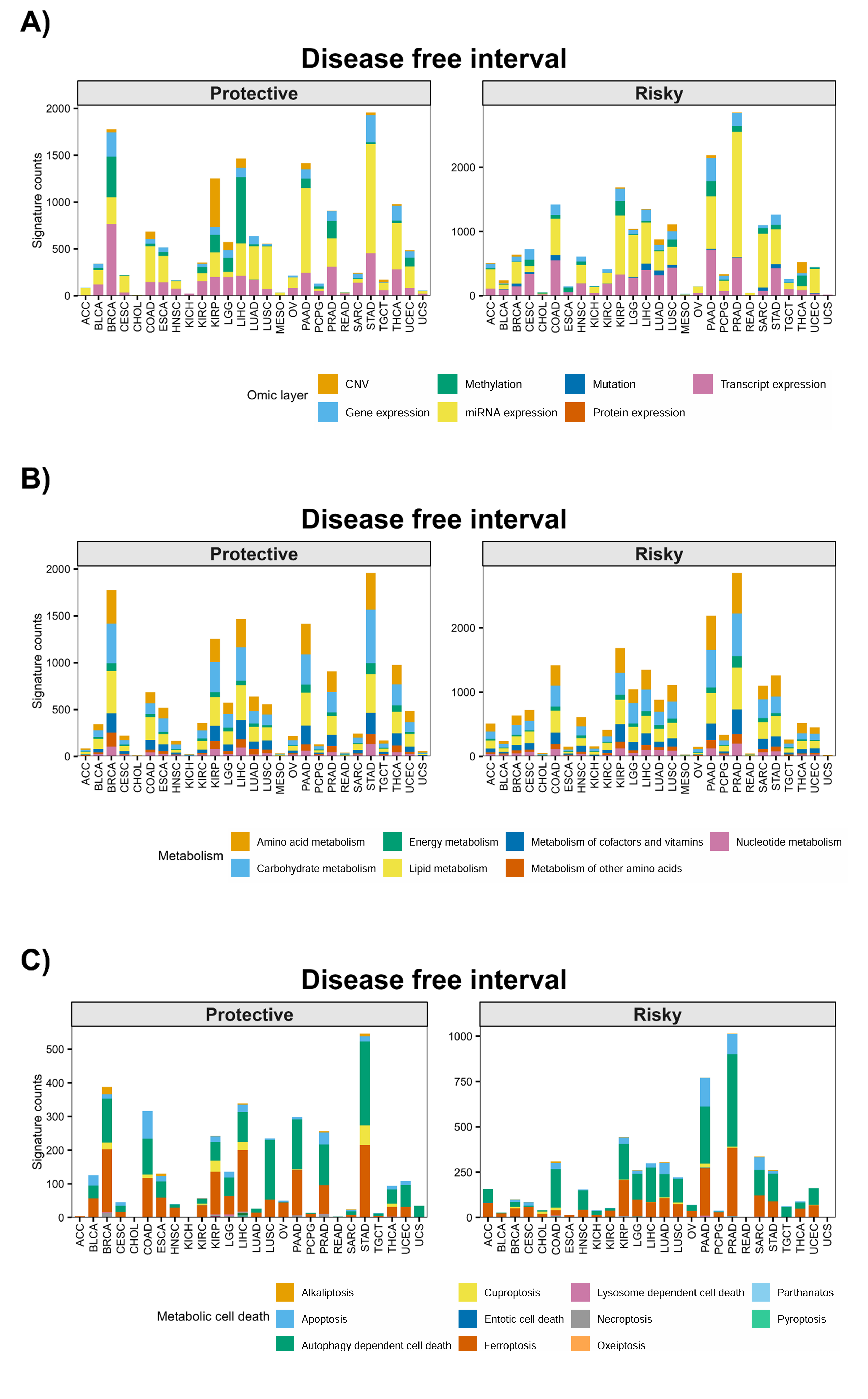

Supplement: Supplementary file 1 [file Image6.tif]

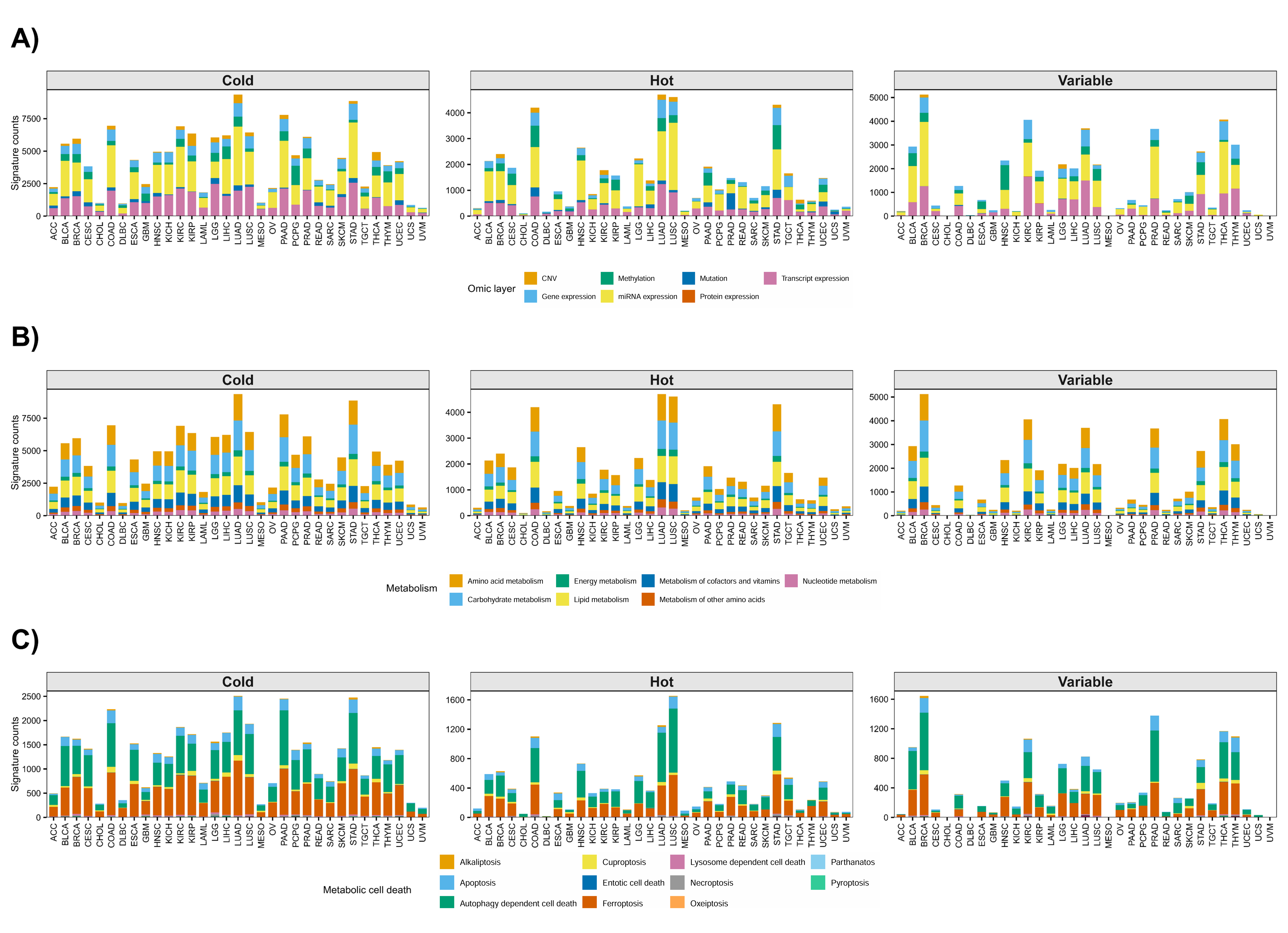

Supplement: Supplementary file 2 [file Image3.tif]

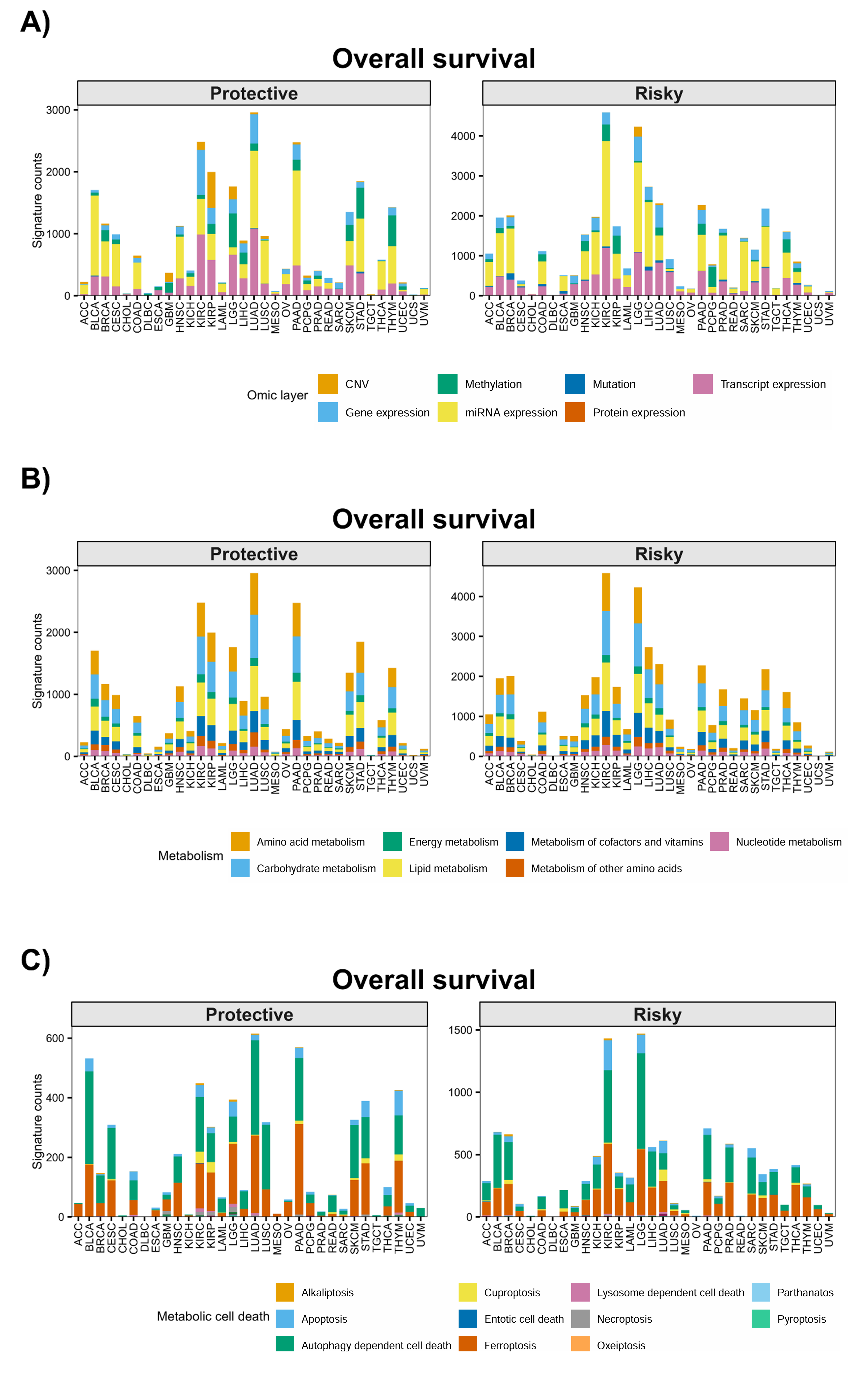

Supplement: Supplementary file 3 [file Image4.tif]

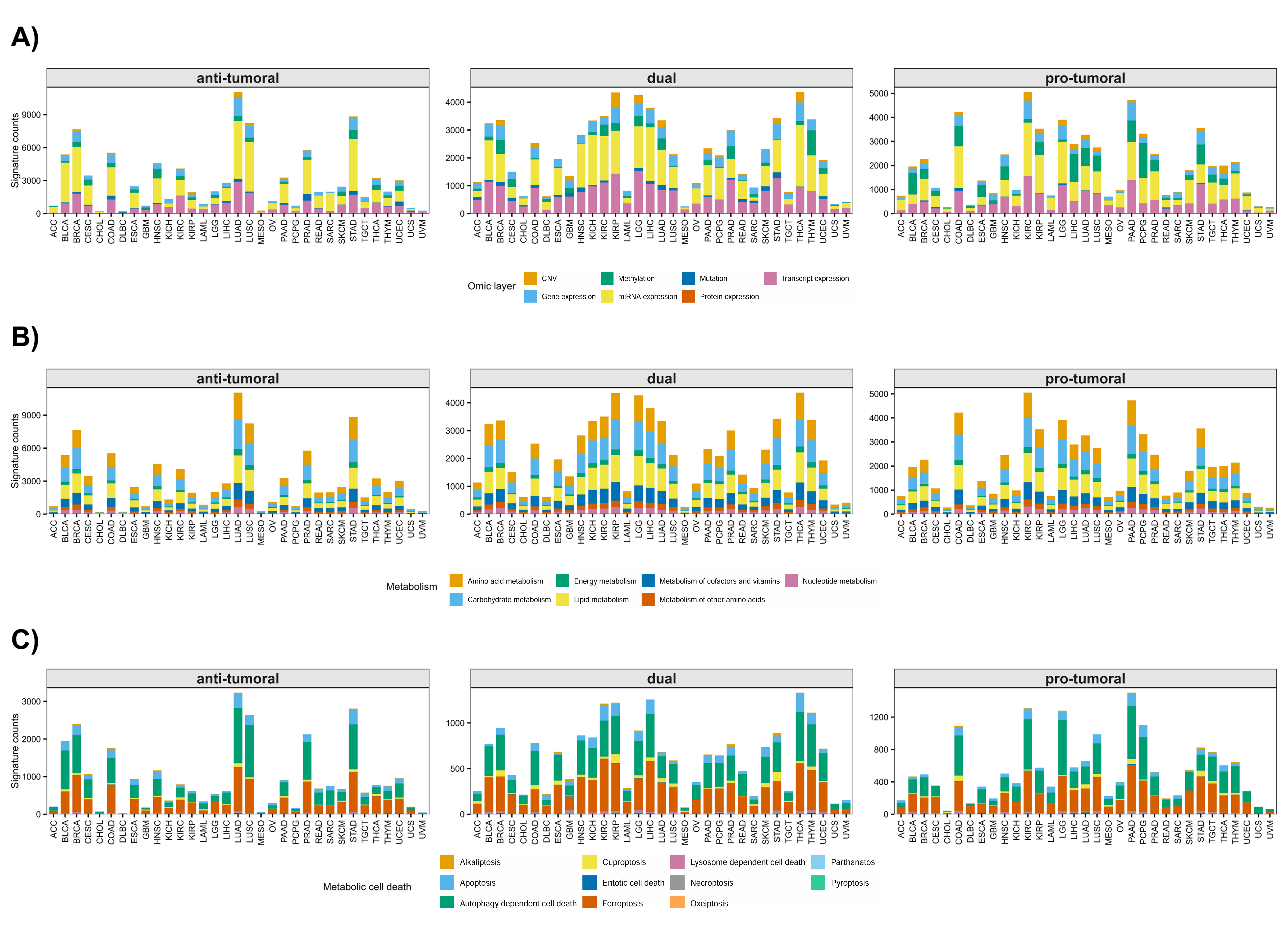

Supplement: Supplementary file 4 [file Image2.tif]

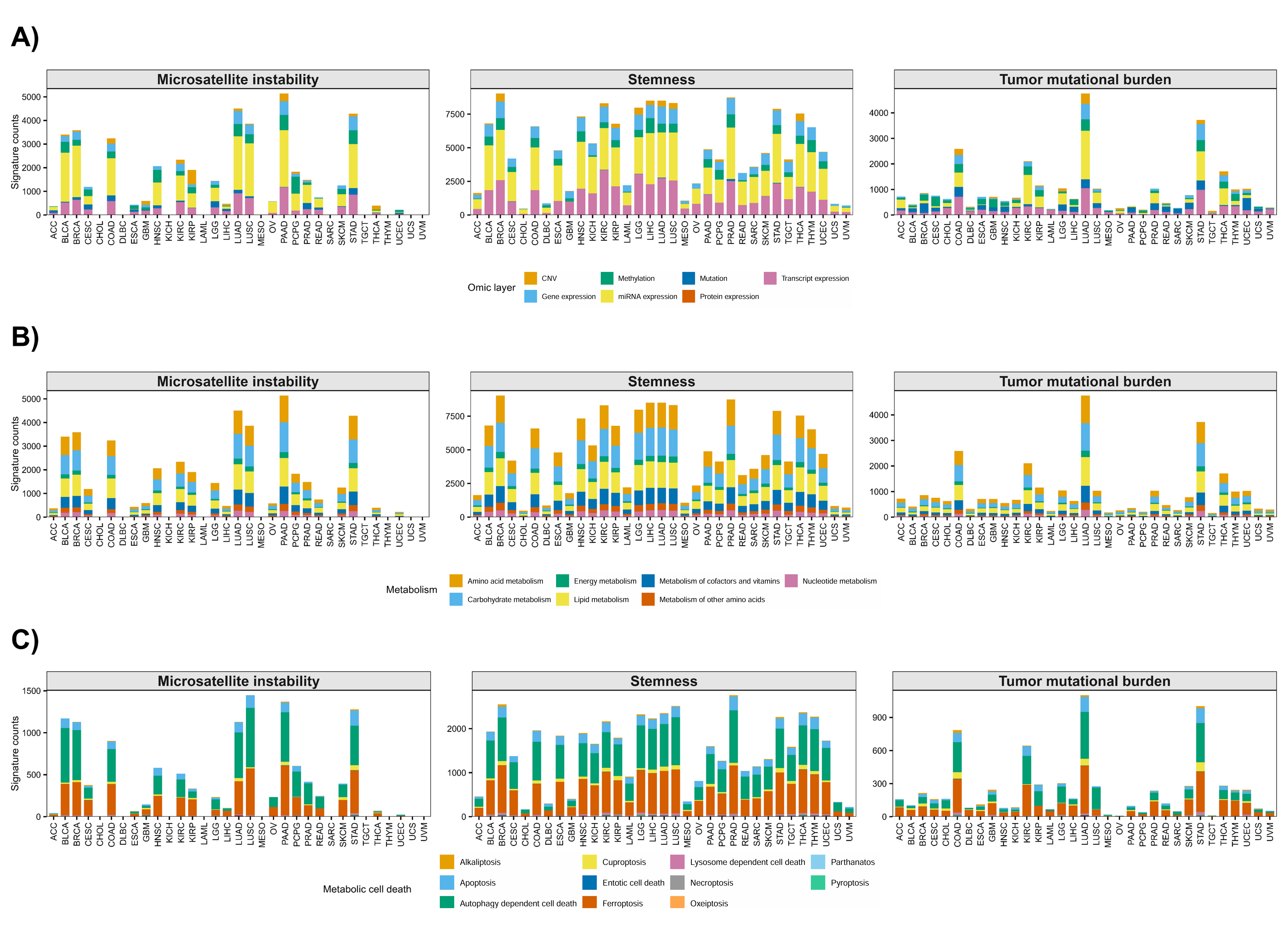

Supplement: Supplementary file 5 [file Image1.tif]

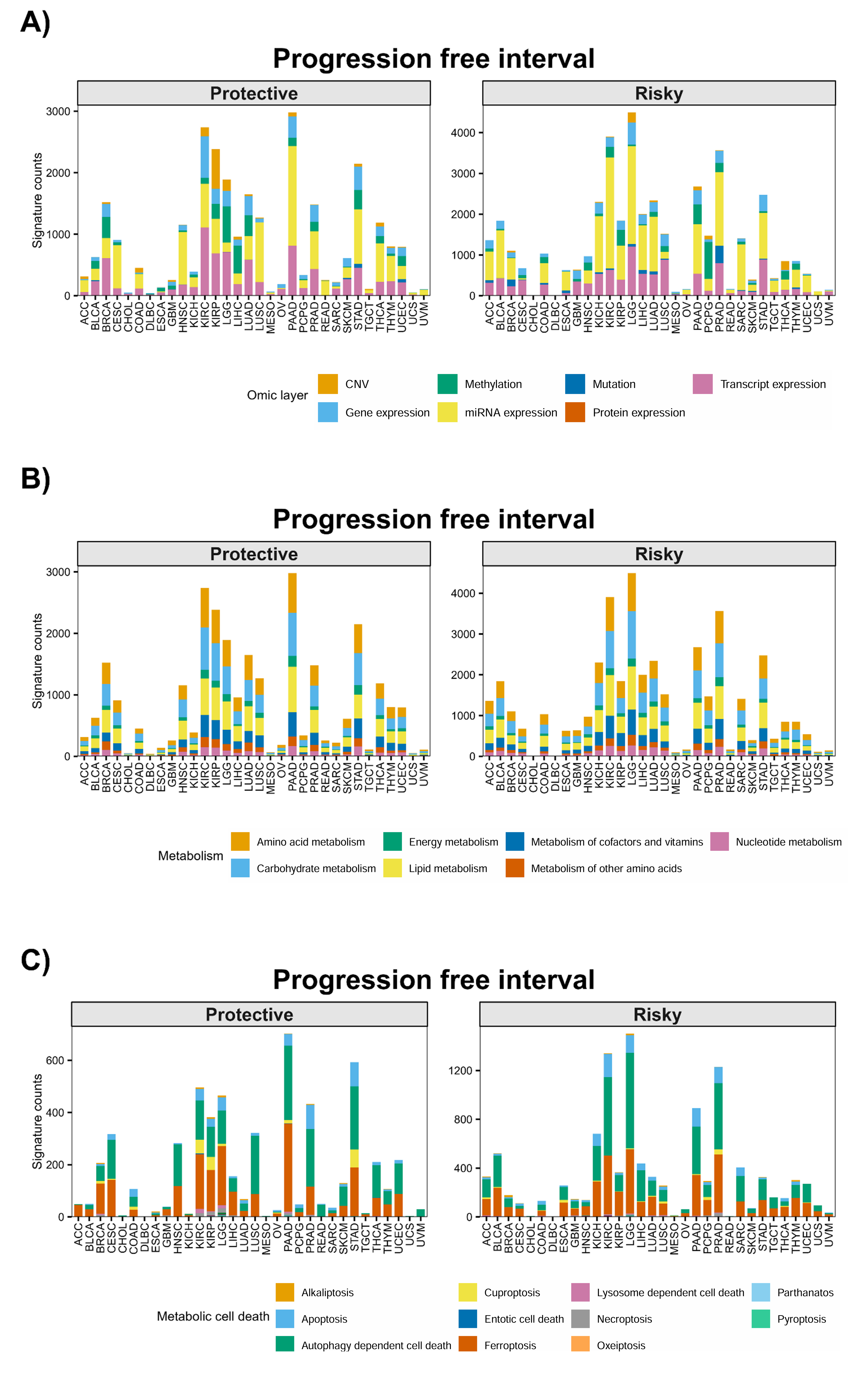

Supplement: Supplementary file 6 [file Image7.tif]

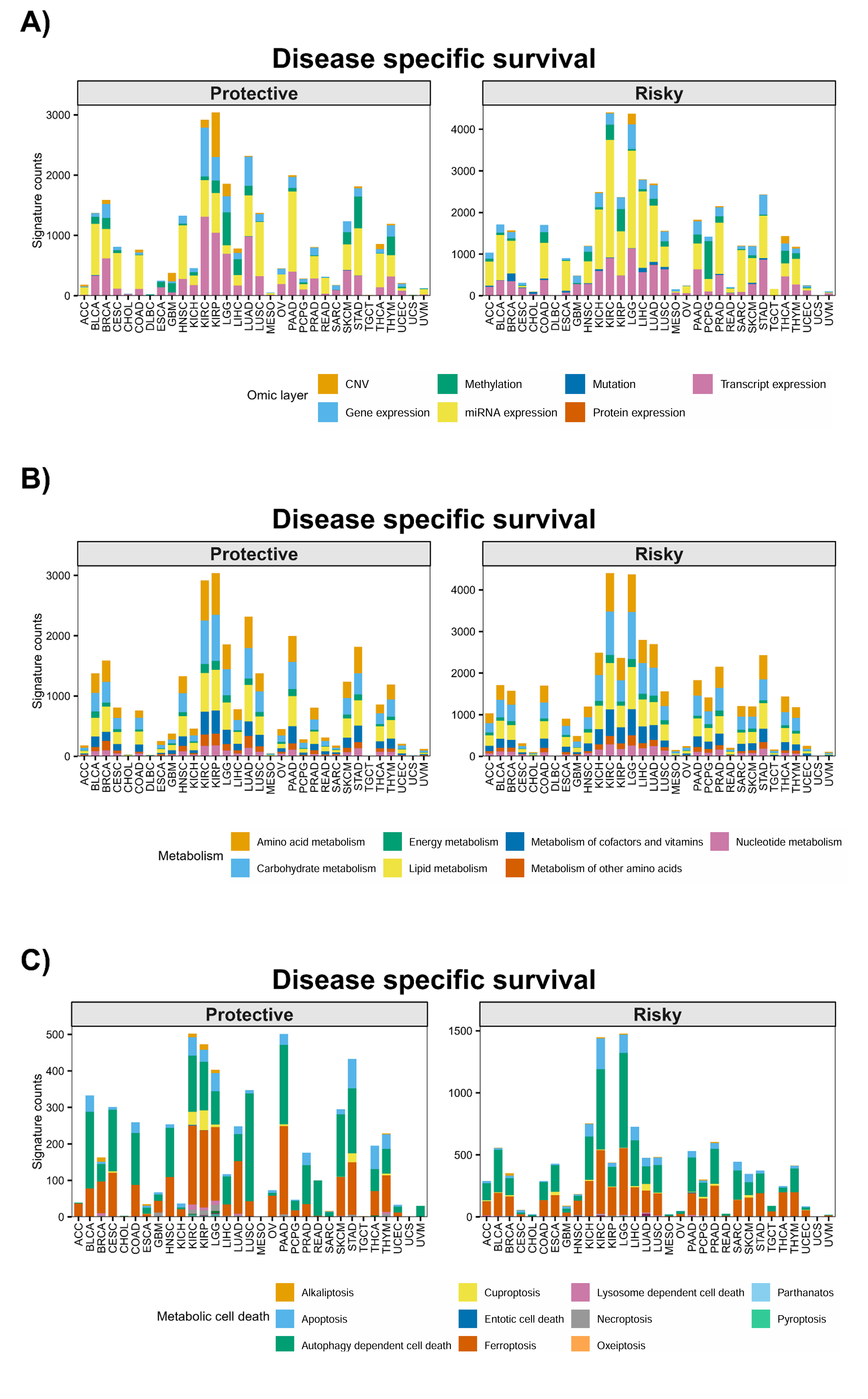

Supplement: Supplementary file 8 [file Image5.tif]
